# Chiral Sulfoxide-Induced Single Turn Peptide α-Helicity

**DOI:** 10.1038/srep38573

**Published:** 2016-12-09

**Authors:** Qingzhou Zhang, Fan Jiang, Bingchuan Zhao, Huacan Lin, Yuan Tian, Mingsheng Xie, Guoyun Bai, Adam M. Gilbert, Gilles H. Goetz, Spiros Liras, Alan A. Mathiowetz, David A. Price, Kun Song, Meihua Tu, Yujie Wu, Tao Wang, Mark E. Flanagan, Yun-Dong Wu, Zigang Li

**Affiliations:** 1 School of Chemical Biology and Biotechnology, Peking University Shenzhen Graduate School, Shenzhen, 518055, China; 2 Cardiovascular and Metabolic Diseases Medicinal Chemistry, Pfizer, Inc., 620 Memorial Drive, Cambridge, MA, 02142, USA; 3 Department of Biology, Southern University of Science and Technology, Shenzhen, China; 4 Center for Chemistry Innovation and Excellence, Pfizer Inc., Eastern Point Road, Groton, CT, 06340, USA; 5 College of Chemistry, Peking University, Beijing, 100871, China

## Abstract

Inducing α-helicity through side-chain cross-linking is a strategy that has been pursued to improve peptide conformational rigidity and bio-availability. Here we describe the preparation of small peptides tethered to chiral sulfoxide-containing macrocyclic rings. Furthermore, a study of structure-activity relationships (SARs) disclosed properties with respect to ring size, sulfur position, oxidation state, and stereochemistry that show a propensity to induce α-helicity. Supporting data include circular dichroism spectroscopy (CD), NMR spectroscopy, and a single crystal X-ray structure for one such stabilized peptide. Finally, theoretical studies are presented to elucidate the effect of chiral sulfoxides in inducing backbone α-helicity.

According to recent statistical analysis of the Protein Data Bank (PDB), roughly 60 percent of protein-protein interactions (PPIs) involve α-helices at the interface, and most of the α-helices consist of 15 amino acids or less^1^,^2^. These PPIs play a critical role in numerous biological processes and represent a rich array of potential therapeutic targets^3^,^4^,^5^. Despite small molecule’s therapeutic potential^6^,^7^,^8^,^9^, development of small molecule PPI ligands is still formidable due to the shallow, large, and even disconnected PPI surfaces^10^; meanwhile, large proteins are often unsuitable for intracellular PPIs due to poor cell permeability. Consequently, intracellular PPIs were once considered “undruggable”.

Short peptides are generally less structurally defined in aqueous solutions as water molecules can disrupt the intramolecular hydrogen bonding of the peptide backbone. When constrained into rigid α-helical conformations, short peptides can mimic protein binding surfaces and exhibit greater resistance against metabolizing enzymes^11^. Developing methodologies to predictably induce α-helices in short peptides is therefore of considerable interest for peptide-based drug development^12^,^13^,^14^,^15^,^16^. Over the past several decades, various approaches, spanning non-covalent and covalent strategies, to reinforcing the bioactive helical conformation were developed^17^. Various non-covalent strategies have been used to stabilize peptide backbone toward the a-helical conformation, including helix-nucleating templates^18^,^19^,^20^,^21^ and introducing α, α-disubstituted amino acid, such as aminoisobutyric acid^22^,^23^. While for covalent strategies, a common approach for inducing and stabilizing fixed secondary structure in peptides is by tethering two side chains on the same face of the helix via different cross-links. These so-called stapled peptides have been extensively studied in recent years and have been the subjects of numerous reviews^24^,^25^,^26^. In general, these stapled peptides have different cross-links, such as aryl, alkenyl, disulphide, “click” triazole, amide, and thioether connectors^16^,^27^,^28^,^29^,^30^,^31^,^32^,^33^,^34^. Besides, while we know that peptides are composed of chiral L-amino acids, the effect of stereocenters within the cross-links in peptide secondary structure has not been extensively studied.

## Results and Discussion

**Figure 1:**
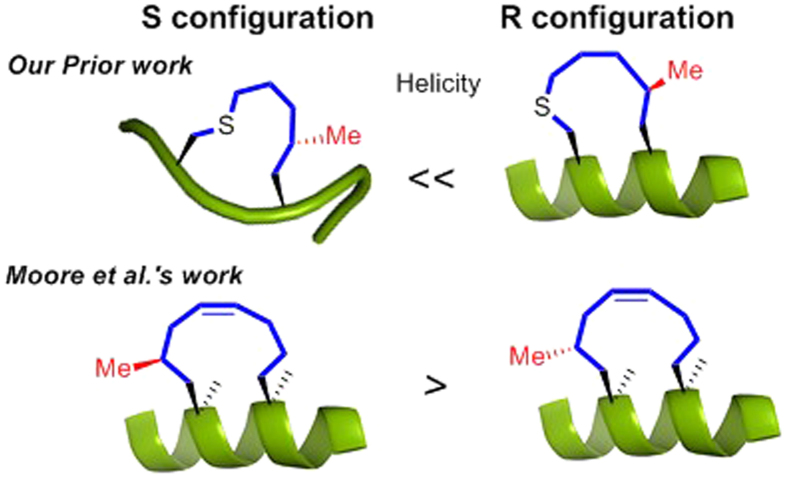
On-tether chiral centres influence the secondary structure of the peptide.

Recently, our group reported a chiral carbon-centered tether that controlled the secondary structure of peptides via its absolute configuration^35^. Meanwhile, Moore *et al*. reported that a chiral center on a stapled peptide affected a peptide’s secondary structure^36^ (Fig. 1). Notably, the preference of different chiral center positions of Moore *et al.’*s system and ours may be caused by the missing of α-methyl groups on the linking amino acid residues in our system and the bonding pattern differences between olefin and single bonded thiolether tethers. In the present study, we set out to evaluate short peptides with chiral sulfoxide-containing cross-links and examined the structure-activity relationships (SARs) that induced α-helicity within these molecules. Comparing with the thiolether tethered peptides, the oxidized peptides showed better solubility in aqueous solution. Notably, experimental and simulation results for the less hindered sulfoxide model elucidate the importance of a precisely positioned chiral center for inducing backbone peptides’ helicity. We discovered that in addition to cross-link length and the position of the sulfoxide moiety, the absolute configuration of the sulfoxide appears to be essential for inducing α-helicity. Importantly, this on-tether chiral-centered-induced α-helicity appears to afford general flexibility in amino acid content for the peptides that were evaluated. The measured enhancement in α-helicity induction is comparable to the highest reported values by CD spectrum, but have a relatively higher fray at C-terminal compared with the amide cross-linked peptides Ac-*c*(1,5)-[KAAAD]-NH_2_ by 2D NMR study^34^,^37^.

**Figure 2:**
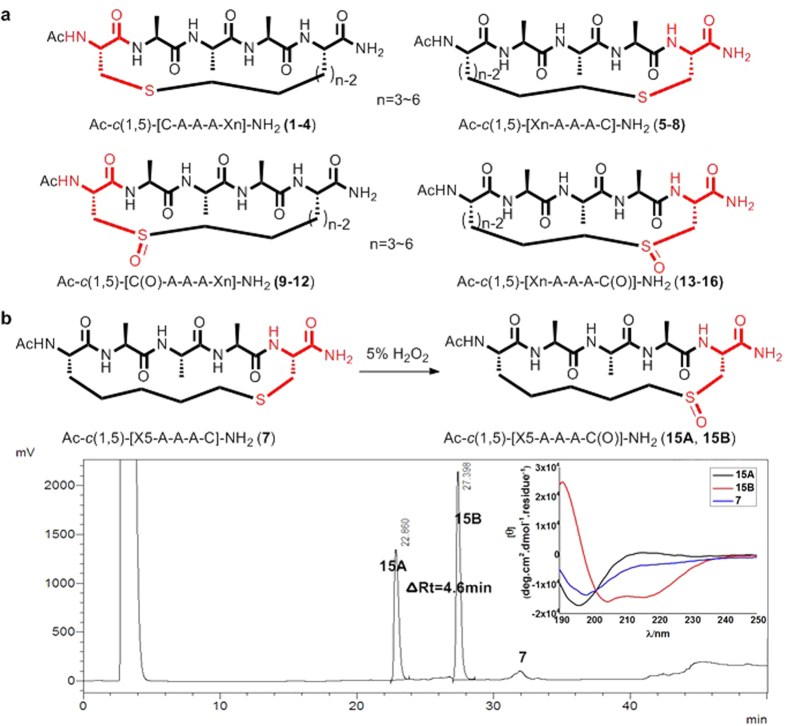
(**a**) Sequence of peptide **1**-**16**, where A is L-alanine, C is cysteine, *c*(x, y) signifies cross-link forming amino acid. (**b**) Oxidation of peptide **7** to (S)-**15A** and (R)-**15B**. HPLC spectrum of the oxidation reaction mixture. CD spectroscopy was performed in 10 mM PBS (pH = 7.4) buffer at 25 °C.

The initial construct of the peptides prepared for this investigation involved pentapeptides of similar composition to those that have appeared in relevant literature^34^. In this study, L-Cysteine and L-amino acids Xn (as shown in SI) with an aliphatic alkenyl modification were chosen as coupling partners^38^. Single turn peptides Ac-*c*(1,5)-[CAAAXn]-NH_2_ (**1**-**4**) and Ac-*c*(1,5)-[XnAAAC]-NH_2_ (**5**-**8**) were picked as simplified model peptides to eliminate possibility of any sequence perturbations (Fig. 2a). Peptides **1**-**8** exhibited only minimal helical character based on circular dichroism (CD) spectroscopy measurements with 10 mM PBS (pH = 7.4) buffer as solvent (SI Fig. 1a)^37^. Peptides **5**-**8** (with cysteine at the C terminus) showed slightly more α-helical content than peptides **1**-**4** (with cysteine at the N terminus) in PBS and TFE buffer, with peptide **7** exhibiting the highest degree of α-helicity in TFE buffer (SI Fig. 1b). Oxidation of these peptides produced a mixture of their corresponding sulfoxide diastereomers, indicated as peptides **9**-**12** and peptides **13**-**16** (Fig. 2b). For peptides **9**, **11** and **12**, the diastereomers were difficult to separate by HPLC, and CD spectroscopy measurements of the mixtures suggested a random coil structure. The diastereomers of **10** were separable and exhibited only minimal α-helicity (SI Fig. 2a). The diastereomers of **13**-**16** were easily separable, suggesting they adopted higher conformational differences in solution. CD spectroscopy measurements of (R)-**15B** (the diastereomer with a longer retention time) consistently show a high degree of α-helicity, while the CD spectrum for (S)-**15A** indicates a random coil (Fig. 2b; see also SI Fig. 2b for CD spectra of peptides **13A**-**16A**). Importantly, the CD spectra for peptides **13B**-**16B** (SI Fig. 2c) suggest that X5 provided the optimal linker length for inducing α-helicity. We also evaluated the influence of chiral sulfoxide on CD spectrum by using (S)-(-)-2-Methyl-2-propanesulfinamide(S-BSN) and its enantiomer R-BSN, which have distinct t-butyl and amino group substitutions on the chiral center. By subtracting R-BSN and S-BSN absorption on (S)-**15A** and (R)-**15B**’s CD spectrum, their α-helical content is somewhat enhanced (base on [θ]_215_) while with a slightly shifted minima ([θ]_203_/[θ]_217_; 1.0:0.73) (SI Fig. 3). Overall, chiral sulfoxide center itself has limited influence on the peptides’ CD sepctra. Furthermore, these data clearly indicate that the C terminus is the optimal position for the cysteine and that shifting the sulfoxide moiety one atom towards the cross-linker center diminishes α-helicity (Ac-*c*(1,5)-[X4AAAC*(O)]-NH_2_ peptide **17**; C*: homocysteine, See SI Fig. 4a for CD spectroscopies of peptide **17**). ^1^H NMR and NOE measurements were taken for (S)-**15A**, (R)-**15B** and (R)-**19B**) (SI Fig. 5, SI Fig. 6). A number of characteristic spectral features of a high degree helical behavior were observed. First of all, conspicuously small ^3^*J*_NH-Hα_ constants (<6 Hz) were observed for all amide resonances except Cys5 for (R)-**15B** (SI Table 1). For (R)-**19B**, the side chain difference on residue 3 resulted in a slightly larger ^3^*J*_NH-Hα_ (7.2 Hz) (SI Table 1). The second NMR evidence for helicity was smaller Δ*δ*/T (<4 ppb/K) for Ala2, Ala4, Cys5 and one C-terminal NH for (R)-**15B**, which suggested propensity of forming intra-molecular hydrogen bonds (SI Table 2). Long range ROEs (NH(i) – Hα(i-3)) were also observed for (R)-**15B**. However, due to the signal overlapping of α protons, it’s hard to confirm the key ROEs from NH(i) - Hα(i-4). Finally, from a practical standpoint, the chiral sulfoxide-containing cross-link approach avoids the need for quaternary carbon centers at the amino acid alpha position. This represents a potential advantage over methodologies requiring such a carbon center in order for olefin metathesis chemistry to proceed^29^,^32^,^39^. Notably, the addition of an α-methyl group to X5 of (R)-**15B** to get its derivative peptide (R)-**18B** (Ac-*c*(1,5)-[X5*AAAC(O)]-NH2, X5*: α methylated X5) greatly diminishes α-helicity (see SI Fig. 4b for CD spectroscopy of (S)-**18A** and (R)-**18B**). Sulfoxide could racemize under 6 M HClO_4_^40^. Under the biorelevant acidic or basic conditions tested, there was no detectable sulfoxide racemization.

**Figure 3:**
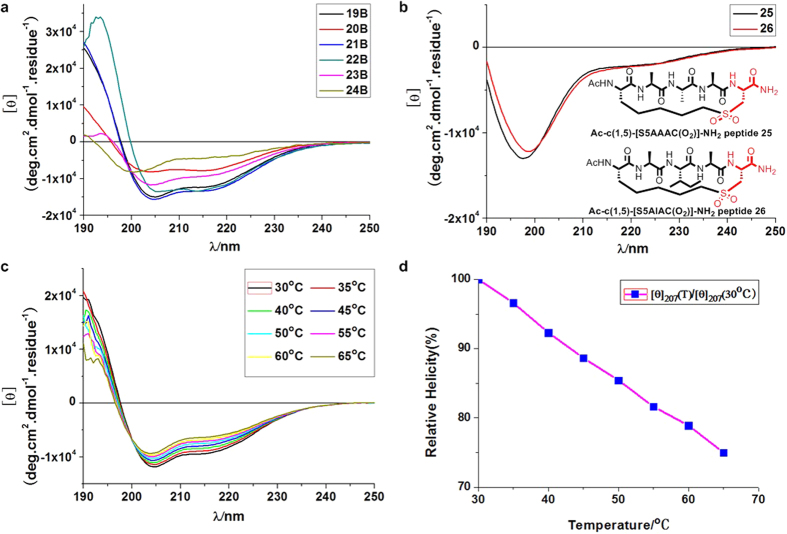
(**a**) CD spectra of (R)-diastereomer **19B-24B**. **(b**) CD spectra of sulfone-containing peptides **25** and **26** show minimal helix contents. (**c**) CD spectroscopy was performed for (R)-**19B** at increasing temperatures. Its helicity decreased slightly as temperature increased (**d**) At 65 °C, (R)-**19B** retained 75% of the helicity that it showed at 30 °C. CD spectroscopy was performed in 10 mM PBS (pH = 7.4) buffer at 25 °C.

Subsequently, the generality of these observations was examined. A series of single turn α-helical peptides with varying sequences and containing the on-tether chiral center as indicated above were synthesized. These peptides were separated by HPLC to give peptides (R)-**19B**-**24B,** and their α-helicities were tested via CD spectroscopy (Fig. 3a). As shown in Table 1, all of these peptides exhibited a high degree of α-helicity, including (R)-**20B,** which contained a flexible glycine. When sulfoxide-containing peptides **15** and **19** were further oxidized to their corresponding sulfones (**25** and **26**), significantly diminished α-helicity was observed (Fig. 3b). This finding suggests that the sulfoxide’s chirality plays a critical role in controlling the peptide backbone’s α-helicity.Additionally, temperature-dependent CD spectroscopy measurements on (R)- diastereomer **19B** indicate temperature tolerance, and at 65 °C, (R)-**19B** retained 75% of the helicity that it showed at 30 °C (Fig. 3c,d).

**Figure 4:**
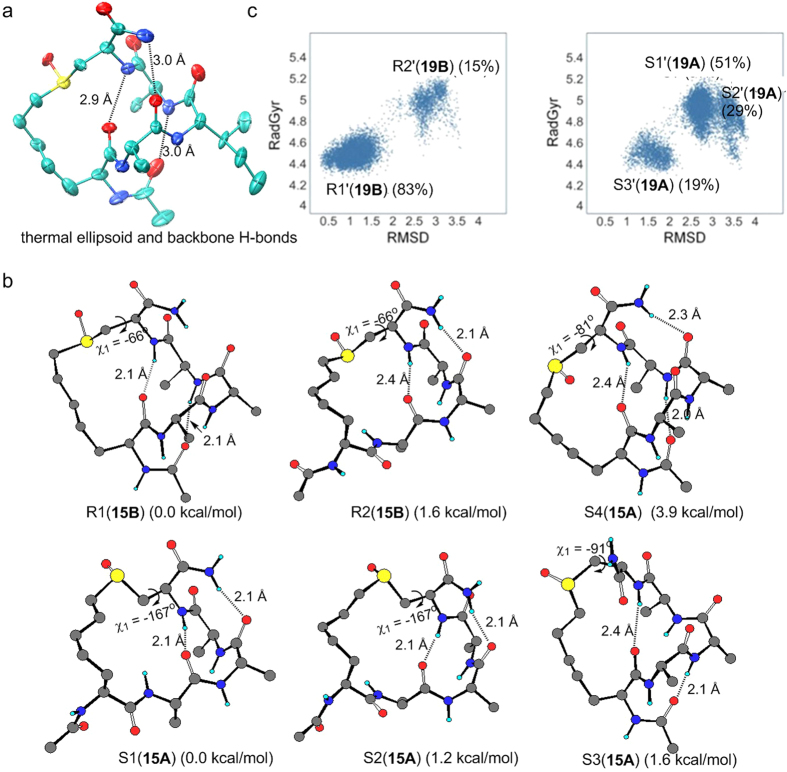
Crystal structure of (R)-**19B** and calculated structures of (S)-**15A**, (R)-**15B**. (**a**) X-ray crystal structure of (R)-**19B** with thermal ellipsoids shown at the 50% probability level with three α-helical hydrogen bonds. (**b**) Conformers of (R)-diastereomer **15B** (R1, R2) and (S)-diastereomer **15A** (S1, S2, S3, S4) with increased stability, calculated with density functional theory at PBE1PBE/6-31 + G** level. Relative free energies are given below each structure. (**c**) Conformations sampled in REMD simulations of (S)-**19A** and (R)-**19B**. The x-axis is the backbone RMSD of the conformations, with respect to the X-ray structure of (R)-**19B**. The y-axis is the radius of gyration of the conformations. The clusters are labelled as R1′(**19B**), R2′(**19B**) and S1′-S3′(**19A**), similar to structures R1(**15B**), R2(**15B**), and S1-S3(**15A**) in (**b**), see SI Fig. 8 for the structures of R1′(**19B**), R2′(**19B**) and S1′-S3′(**19A**).

Finally, X-Ray crystallography of (R)-**19B** confirmed the aforementioned CD and NMR data and unambiguously established the absolute (R) configuration for the sulfoxide (S=O) chiral center. Furthermore, the crystal structure clearly indicates that there is no hydrogen bond between the sulfoxide oxygen and any of the peptide backbone hydrogen bond donors, and that the intramolecular hydrogen-bonding pattern agrees with the proposed α-helix model (Fig. 4a). The small C-S-C bond angle at the sulfoxide (S=O) center measures 96°, which helps to constrain the peptide backbone (SI Fig. 9). By contrast, the (S)-**19A** could not be crystallized. In the crystal structure of (R)-**19B**, two nearly identical structures co-exist in one crystal lattice. In addition, the presence of water molecules within the crystal lattice suggests the peptide retains its helical conformation in solution. The three residues in the middle are very close to being an ideal α-helix with average rise-per-residue of 1.47 Å and 3.68 residues per turn, and the backbone dihedral angles (φ, ψ) of residues X5-1 to Ala-4 were very close to regular α-helical dihedral angles (SI Table 3). The dihedral angles of the C-terminal residue Cys-5 (φ = −109°, ψ = −8°) deviate significantly from those of a perfectly regular α-helix^41^ (φ = −65°, ψ = −40°). This observation is very similar to that seen in crystal structures of hydrogen bond surrogate (HBS)-stabilized α-helixes^42^ and protein crystal structures^41^.

Further theoretical studies were carried out using (S)-**15A** and (R)-**15B** as models. Their possible conformations were researched using HyperChem software^43^. Low energy conformations were further optimized with the density functional theory (DFT) method of PBE1PBE^44^, and the SMD model^45^ was used to estimate the solvent effect of water. Free energies were obtained via vibrational frequency calculations. For the (R)-**15B**, the most stable conformer was found to be α-helical as indicated in structure R1(**15B**) (Fig. 4b). This is very similar to what was observed in the crystal structure of (R)-**19B**. R2(**15B**), with a 3_10_-helix (two successive β-turns) was found to be less stable than R1 by about 1.2 kcal/mol. However, the two most stable conformers (S1(**15A**), S2(**15A**)) of the (S)-diastereomer (**15A**) are not α-helical, instead they contain a short 3_10_-helical structure and two extended residues, which consistent with the NMR result of (S)-**15A**. A distorted α-helical structure S3(**15A**) is 1.6 kcal/mol less stable than S1(**15 A**). As shown in Fig. 4c, these results are in good agreement with the replica-exchange molecular dynamics (REMD)^46^ simulation (with AMBER99SB/GB^47^,^48^ force field) results for (S)-**19A** and (R)-**19B**, where R1′(**19B**) and S1′(**19A**), which correspond to R1(**15B**) and S1(**15A**), respectively, are dominant.

A question remains: why is the helical structure of the (S)-diastereomer disfavored? In the α-helical structure R1(**15B**), the χ1 (N-C-C-S) dihedral angle is approximately −66°. The S=O bond points away from the peptide α-helix so that there is no steric hindrance. We optimized a structure S4(**15A**) that is nearly identical to R1(**15B**) except that the sulfoxide center is in (S) configuration. S4(**15A**) is calculated to be less stable than S1(**15A**) by about 3.9 kcal/mol. This is due to the steric interactions between the S=O oxygen atom and the peptide as indicated by the increase in χ1 to −81°. One way to avoid the steric interactions is to rotate the χ1 dihedral angle so that the S=O is away from the peptide. This is the case in S1(**15A**) and S2(**15A**), where χ1 changes to around −167°. However, this change renders the linker too short to maintain the α-helix. As a result, the N-terminus is forced away from the α-helical structure and only a short 3_10_-helix remains in S1(**15A**) and S2(**15A**). Another way to avoid the steric interactions in S4(**15A**) is to distort the α-helix, as is the case in S3(**15A**). However, this causes significant destabilization.

## Conclusion

This study demonstrates that a precisely-positioned sulfoxide moiety in a peptide “staple”, with (R)-stereochemical configuration, is capable of inducing a single turn peptide into α-helical structure in aqueous solution. CD spectra and corroborating NMR experiments support these observations. By contrast, the corresponding peptides containing thioethers and sulfones exhibited minimal enhancement of α-helicity. α-helicity critically depends on the cross-link (staple) length, for which seven atoms are optimal. DFT and REMD calculations also show that peptides with an (R)-sulfoxide cross-link favor α-helical conformation, while the (S)-containing peptides do not. This stabilization strategy exhibits excellent peptide sequence tolerance. Finally, this investigation into the ability of (R)-sulfoxides to induce α-helical conformation in peptides informs research regarding the chiral modification of cross-links in staple peptides.

## Methods

### Preparation of thiolether, sulfoxide and sulfone cross-linking peptides

Thiolether cross linking peptide **1** and **2** was synthesized following general procedure A in supporting information. First H_2_N-Ala-Ala-Ala-Xn-resin (Rink amide MBHA, n = 3, 4) was synthesized using Fmoc chemistry. Then thiol-addition of N-Acetyl-L-cysteine to the terminal olefin of resin bond Xn was conducted by DMPA catalyst and UV irradiation. The result peptide H_2_N-Ala-Ala-Ala-Xn(N-Acetyl-L-cysteine)-NH_2_ was cleaved by cleavage cocktail (TFA/TIS/EDT/H2O 94/1/2.5/2.5) from resin, and then macro-cyclized in solution by amide bond formation. Peptides **3**, **4**, **7**, **8** and the thiolether counterpart for peptide **17**-**24** was synthesized following our former study^35^, and detailed procedure was presented in the supporting information (general procedure B). Peptide **5** and **6** was synthesized with similar procedure as **1** and **2**, except that CTC resin and L-Cysteinamide monohydrochloride was used instead of Rink amide AM resin and N-Acetyl-L-cysteine.

The sulfoxide cross linking peptide was produced by oxidation of their thiolether counterpart. Oxidation reaction was conducted in 5% H_2_O_2_ (1 mL per 2.5 mg thiolether peptide) for 3 h at room temperature (1% H_2_O_2_ and ice bath for **23** and **24**).

Sulfone cross linking peptide **25** and **26** was synthesized by oxidizing sulfoxide peptide **15** and **19** with 1.5% H_2_O_2_ in acetic acid (1 mL per 3 mg peptide) for 8 h at room temperature.

### Characterization of Peptides by CD spectroscopy

Stapled peptides were dissolved in aqueous 10 mM potassium phosphate solution (pH 7.4) or 50% TFE buffer to concentrations of 50–800 μM. CD spectra were obtained on a Chirascan Circular Dichroism Spectrometer at 25 °C using the following standard measurement parameters: wavelength, 190–250 nm; step resolution, 0.5 nm; speed, 20 nm/sec; accumulations, 10; response, 1 sec; bandwidth, 1 nm; path 3 length, 0.1 cm. Every sample was scanned twice and the final CD spectrum was smoothed to reduce noise. The α-helical content of each peptide was calculated by dividing the mean residue ellipticity [θ]_222obs_ by the reported [θ]_222obs_ for a model α-helical pentapeptide.

### Crystallization and Data Collection

Peptide **19B** was dissolved in 50% CH_3_OH with 10 mg/mL and crystallized at 25 °C using sitting drop vapor diffusion method against reservoir solution of 90% CH_3_OH. Crystals appeared, attained full size within 20 days, and then started to decay after four weeks. Crystals were flash-frozen in liquid nitrogen and cryo-protected by 30% Glycerol in mother liquor. The crystals were screened and collected at 100 K by in-house X-ray diffraction system equipped with high-intensity sealed Copper tube X-ray generator (Rigaku^®^ MicroMax-002+), an AFC11 goniometer, a Saturn 944+ CCD detector (Rigaku^®^) and an Oxford Cryo-system.

### NMR data acquisition and processing

Peptides were prepared in 1 × PBS buffer containing 10% D_2_O or PBS/TFE (50/50 v/v) within a concentration range of 1–5 mM. 0.5 mM sodium (3-trimethylsilyl)-2, 2, 3, 3-tetradeuterio-propionate (TSP) was added for 1 H chemical shift reference. NMR data was recorded on Bruker AVANCE III 500 MHz spectrometer. 1D spectra were acquired using excitation sculpting for water suppression. 32 K data points were collected with 8000 Hz sweep width, 3 s repetition time and 16 scans. The temperature ranged between 283 K and 303 K for temperature coefficient measurement. Standard 2D NMR methods were used to make assignments of proton signals. TOCSY (mixing time 80 ms) and ROESY(relaxation delay 2.0 s, mixing time 200 ms) experiments were recorded at 283 K in phase-sensitive mode with spectral width of 5000 Hz, 2048 points on F2 and 256 increments on F1. Solvent suppression was achieved using water gate W5 method. NMR data were processed using Topspin 3.0. We used the temperature coefficient as a tool to characterize the propensity of exchangeable protons to form IMHBs. The protection of IMHBs decreased the temperature dependence of the chemical shift of the exchangeable protons. This resulted in a smaller value of Δδ/T compared to the non-IMHB donors. In general, the cutoff value for Δδ/T for IMHBs was solvent dependent. In aqueous solution, values of Δδ/T less negative than −4.0 ppb/K usually indicated hydrogen bonding. Peptide characterization 1HNMR was performed in DMSO-*d*^6^ or in H_2_O/D_2_O 9:1.

### Quantum Mechanical Calculations

To generate initial structures for further QM calculations, conformational searches were carried out with Amber and Charmm force fields and high temperature Monte Carlo simulations (MC, 300 K–2000 K, 105 steps) implemented in the HyperChem software. The solvent effect was treated implicitly using distance-dependent dielectric constants. The obtained structures were separated according to the chirality of the sulfoxide center (S- or R- peptides). For each diastereomer, the top 200 structures from each force field were selected and all 400 structures were optimized using density functional theory (DFT) method of PBE1PBE with 6–31 G* basis set. The popular PBE1PBE (also called PBE0) functional was found to be among the best for describing non-covalent interactions, especially H-bonding^48^. After removing nearly identical ones in the pool of optimized structures, the remaining structures within 5 kcal/mol were further optimized using PBE1PBE/6–31 + G**. All QM calculations were performed using Gaussian 09 software^48^.

### Molecular Dynamics Simulations

Simulations were performed using AMBER10 software package with AMBER99SB^43^ force field, a modified Generalized Born solvation^44^ model. The missing force field parameters of the linkers were obtained from GAFF forcefield, and partial charges were calculated using Jaguar. All non-bonded interactions were evaluated at every 1.0 fs time step. The temperature in each simulation was kept constant using Langevin dynamics with the collision frequency of 1.0 ps-1. The initial structures were alpha-helices with linkers built by Maestro. To remove the bias of the initial structure, each peptide was simulated using regular MD for 10 ns. The final structure of the simulation was used for replica exchange MD (REMD)^46^ simulations. The conformations of the peptides were generated using REMD simulation method implemented in AMBER10. Eight replicas of the system were simulated simultaneously at temperatures 270, 300, 334, 372, 414, 461, 513, 571 K. At intervals of 10 ps, exchanges were attempted among conformations of the replicas at neighboring temperatures. The REMD simulations were carried out for 20 ns (a total of 160 ns simulation) and 20,000 snapshots were saved for each temperature. Conformations collected at 300 K were used for further analysis.

## Additional Information

**How to cite this article**: Zhang, Q. *et al*. Chiral Sulfoxide-Induced Single Turn Peptide α-Helicity. *Sci. Rep.* **6**, 38573; doi: 10.1038/srep38573 (2016).

**Publisher's note:** Springer Nature remains neutral with regard to jurisdictional claims in published maps and institutional affiliations.

## Supplementary Material

Supplementary Information

## Figures and Tables

**Table 1:** Helicity measurements for peptides with different sequences and molar ellipticities ([θ]/deg.cm^2^.dmol^−1^.residue^−1^)^37^.

Peptide	Sequence	[θ]_215_	[θ]_207_	[θ]_190_	[θ]_215_/[θ]_207_	Helicity(%)
(R)-**15B**	Ac-*c*(1,5)-[X5AAAC(O)]-NH_2_	−14351	−14763	24129	0.97	100
(R)-**19B**	Ac-*c*(1,5)-[X5AIAC(O)]-NH_2_	−12334	−14211	25560	0.88	86
(R)-**20B**	Ac-*c*(1,5)-[X5AGAC(O)]-NH_2_	−7839	−7731	9570	1.01	54
(R)-**21B**	Ac-*c*(1,5)-[X5AQAC(O)]-NH_2_	−13358	−14886	27175	0.91	93
(R)-**22B**	Ac-*c*(1,5)-[X5AFAC(O)]-NH_2_	−13235	−13379	25940	0.96	92
(R)-**23B**	Ac-*c*(1,5)-[X5ASAC(O)]-NH_2_	−19357	−10950	1610	0.85	65
(R)-**24B**	Ac-*c*(1,5)-[X5KAEC(O)]-NH_2_	−4497	−5552	1975	0.81	31

CD spectroscopy was performed in 10 mM PBS (pH = 7.4, 25 °C). [θ] was calculated according to literature^35^; (R)-**15B**’s helicity was set to 100%, and the helicity of the other peptides were calculated by [θ]_215_/[θ]_215_(**15B**).

## References

[b1] Jochim A. L. & Arora P. S. Assessment of helical interfaces in protein-protein interactions. Mol. BioSyst. 5, 924–926 (2009). 19668855 10.1039/b903202a PMC4575789

[b2] Jochim A. L. & Arora P. S. Systematic analysis of helical protein interfaces reveals targets for synthetic inhibitors. ACS Chem. Biol. 5, 919–923 (2010). 20712375 10.1021/cb1001747 PMC2955827

[b3] Chène P. Drugs Targeting Protein–Protein Interactions. ChemMedChem 1, 400–411 (2006). 16892375 10.1002/cmdc.200600004

[b4] Modell A. E., Blosser S. L. & Arora P. S. Systematic Targeting of Protein–Protein Interactions. Trends Pharmacol. Sci. 37, 702–713 (2016). 27267699 10.1016/j.tips.2016.05.008 PMC4961577

[b5] Phillips C. . Design and structure of stapled peptides binding to estrogen receptors. J. Am. Chem. Soc. 133, 9696–9699 (2011). 21612236 10.1021/ja202946k

[b6] Arkin M. R. & Wells J. A. Small-molecule inhibitors of protein–protein interactions: progressing towards the dream. Nat. Rev. Drug Discov. 3, 301–317 (2004). 15060526 10.1038/nrd1343

[b7] Arkin M. R., Tang Y. & Wells J. A. Small-Molecule Inhibitors of Protein-Protein Interactions: Progressing toward the Reality. Chem. Biol. 21, 1102–1114 (2014). 25237857 10.1016/j.chembiol.2014.09.001 PMC4179228

[b8] Thiel P., Kaiser M. & Ottmann C. Small-molecule stabilization of protein-protein interactions: an underestimated concept in drug discovery? Angew. Chem. Int. Ed. 51, 2012–2018 (2012). 10.1002/anie.201107616 22308055

[b9] Scott D. E., Bayly A. R., Abell C. & Skindmor J. Small molecules, big targets: drug discovery faces the protein-protein interaction challenge. Nat. Rev. Drug Discov. 15, 533–550 (2016). 27050677 10.1038/nrd.2016.29

[b10] Yin H. & Hamilton A. D. Strategies for targeting protein-protein interactions with synthetic agents. Angew. Chem. Int. Ed. 44, 4130–4163 (2005). 10.1002/anie.200461786 15954154

[b11] Bird G. H., Crannell W. C. & Walensky L. D. Chemical synthesis of hydrocarbon-stapled peptides for protein interaction research and therapeutic targeting. Curr. Prot. Chem. Biol. 3, 99–117 (2011). 10.1002/9780470559277.ch110042 PMC4879976 23801563

[b12] Walensky L. D. & Bird G. H. Hydrocarbon-stapled peptides: principles, practice, and progress. J. Med. Chem. 57, 6275–6288 (2014). 24601557 10.1021/jm4011675 PMC4136684

[b13] Spokoyny A. M. . A perfluoroaryl-cysteine S(N)Ar chemistry approach to unprotected peptide stapling. J. Am. Chem. Soc. 135, 5946–5949 (2013). 23560559 10.1021/ja400119t PMC3675880

[b14] Blackwell H. E. & Grubbs R. H. Highly Efficient Synthesis of Covalently Cross-Linked Peptide Helices by Ring-Closing Metathesis. Angew. Chem. Int. Ed. 37, 3281–3284 (1998). 10.1002/(SICI)1521-3773(19981217)37:23<3281::AID-ANIE3281>3.0.CO;2-V 29711420

[b15] Marqusee S. & Baldwin R. L. Helix stabilization by Glu−…Lys+ salt bridges in short peptides of de novo design. Proc. Natl Acad. Sci. USA 84, 8898–8902 (1987). 3122208 10.1073/pnas.84.24.8898 PMC299658

[b16] Meyer F.-M. . Biaryl-bridged macrocyclic peptides: conformational constraint via carbogenic fusion of natural amino acid side chains. J. Org. Chem. 77, 3099–3114 (2012). 22352804 10.1021/jo202105v

[b17] Bock J. E., Gavenonis J. & Kritzer J. A. Getting in shape: controlling peptide bioactivity using conformational constraints. ACS Chem. Biol. 8, 488–499 (2013). 23170954 10.1021/cb300515u PMC4847942

[b18] Kemp D. S. Peptidomimetics and the template approach to nucleation of beta-sheets and alpha-helices in peptides. Trends Biotechnol. 8, 249–255 (1990). 1366733 10.1016/0167-7799(90)90187-3

[b19] Mahon A. B. & Arora P. S. End-Capped α-Helices as Modulators of Protein Function. Drug Discov. Today Technol. 9, e57–e62 (2012). 22712023 10.1016/j.ddtec.2011.07.008 PMC3375709

[b20] Fremaux J. . α-Peptide–Oligourea Chimeras: Stabilization of Short α-Helices by Non-Peptide Helical Foldamers. Angew. Chem. Int. Ed. 54, 9816–9820 (2015). 10.1002/anie.201500901 26136402

[b21] Hoang H. N. . Helix Nucleation by the Smallest Known α-Helix in Water. Angew. Chem. Int. Ed. 55, 8275–8279 (2016). 10.1002/anie.201602079 27226426

[b22] García-Echeverría C., Chène P., Blommers M. J. & Furet P. Discovery of potent antagonists of the interaction between human double minute 2 and tumor suppressor p53. J. Med. Chem. 43, 3205–3208 (2000). 10966738 10.1021/jm990966p

[b23] Banerjee R., Basu G., Chène P. & Roy S. Aib-based peptide backbone as scaffolds for helical peptide mimics. J. Pept. Res. 60, 88–94 (2002). 12102721 10.1034/j.1399-3011.2002.201005.x

[b24] Mullard A. Protein-protein interaction inhibitors get into the groove. Nature Rev. Drug Discov. 11, 173–175 (2012). 22378255 10.1038/nrd3680

[b25] Estieu-Gionnet K. & Guichard G. Stabilized helical peptides: overview of the technologies and therapeutic promises. Expert Opin. Drug Discov. 6, 937–963 (2011). 22646216 10.1517/17460441.2011.603723

[b26] Craik D. J., Fairlie D. P., Liras S. & Price D. The future of peptide-based drugs. Chem. Biol. Drug Des. 81, 136–147 (2013). 23253135 10.1111/cbdd.12055

[b27] Kutchukian P. S., Yang J. S., Verdine G. L. & Shakhnovich E. I. All-atom model for stabilization of alpha-helical structure in peptides by hydrocarbon staples. J. Am. Chem. Soc. 131, 4622–4627 (2009). 19334772 10.1021/ja805037p PMC2735086

[b28] Muppidi A., Wang Z., Li X., Chen J. & Lin Q. Achieving cell penetration with distance-matching cysteine cross-linkers: a facile route to cell-permeable peptide dual inhibitors of Mdm2/Mdmx. Chem. Commun. 47, 9396–9398 (2011). 10.1039/c1cc13320a PMC5428881 21773579

[b29] Schafmeister C. E., Po J. & Verdine G. L. An All-Hydrocarbon Cross-Linking System for Enhancing the Helicity and Metabolic Stability of Peptides. J. Am. Chem. Soc. 122, 5891–5892 (2000).

[b30] Jackson D. Y., King D. S., Chmielewski J., Singh S. & Schultz P. G. General approach to the synthesis of short alpha-helical peptides. J. Am. Chem. Soc. 113, 9391–9392 (1991).

[b31] Lau Y. H. . Functionalised staple linkages for modulating the cellular activity of stapled peptides. Chem. Sci. 5, 1804–1809 (2014).

[b32] Zhang Q., Shi X., Jiang Y. & Li Z. Influence of α-methylation in constructing stapled peptides with olefin metathesis. Tetrahedron 70, 7621–7626 (2014).

[b33] Tian Y. . Stapling of unprotected helical peptides via photo-induced intramolecular thiol–yne hydrothiolation. Chem. Sci. 7, 3325–3330 (2016). 29997825 10.1039/c6sc00106h PMC6006495

[b34] de Araujo A. D. . Comparative α-helicity of cyclic pentapeptides in water. Angew. Chem. Int. Ed. 53, 6965–6969 (2014). 10.1002/anie.201310245 24828311

[b35] Hu K. . An in-tether chiral center modulates peptides’ helicity, cell permeability and target binding affinity. Angew. Chem. Int. Ed. 55, 8013–8017 (2016). 10.1002/anie.201602806 27167181

[b36] Speltz T. E. . Stapled Peptides with γ-Methylated Hydrocarbon Chains for the Estrogen Receptor/Coactivator Interaction. Angew. Chem. Int. Ed. 55, 4252–4255 (2016). 10.1002/anie.201510557 PMC4964982 26928945

[b37] Shepherd N. E., Hoang H. N., Abbenante G. & Fairlie D. P. Single turn peptide alpha helices with exceptional stability in water. J. Am. Chem. Soc. 127, 2974–2983 (2005). 15740134 10.1021/ja0456003

[b38] Aimetti A. A., Shoemaker R. K., Lin C. C. & Anseth K. S. On-resin peptide macrocyclization using thiol-ene click chemistry. Chem. Commun. 46, 4061–4063 (2010). 10.1039/c001375g PMC3969419 20379591

[b39] Boal A. K. . Facile and E-selective intramolecular ring-closing metathesis reactions in 3(10)-helical peptides: a 3D structural study. J. Am. Chem. Soc. 129, 6986–6987 (2007). 17497781 10.1021/ja071148m

[b40] Modena G., Quintily U. & Scorrano G. Novel route to racemization of sulfoxides. J. Am. Chem. Soc. 94, 202–208 (1972).

[b41] Walther D. & Cohen F. E. Conformational attractors on the Ramachandran map. Acta Crystallogr. D 55, 506–517 (1999). 10089363 10.1107/s0907444998013353

[b42] Liu J., Wang D., Zheng Q., Lu M. & Arora P. S. Atomic structure of a short alpha-helix stabilized by a main chain hydrogen-bond surrogate. J. Am. Chem. Soc. 130, 4334–4337 (2008). 18331030 10.1021/ja077704u

[b43] HyperChem^(TM)^ Release 8.0.9 for Windows. Hypercube Inc., Gainesville, FL. http://www.hyper.com/ (2011).

[b44] Adamo C. & Barone V. Toward reliable density functional methods without adjustable parameters: The PBE0 model. J. Chem. Phys. 110, 6158–6170 (1999).

[b45] Marenich A. V., Cramer C. J. & Truhlar D. G. Universal solvation model based on solute electron density and on a continuum model of the solvent defined by the bulk dielectric constant and atomic surface tensions. J. Phys. Chem. B 113, 6378–6396 (2009). 19366259 10.1021/jp810292n

[b46] Mitsutake A., Sugita Y. & Okamoto Y. Generalized-ensemble algorithms for molecular simulations of biopolymers. Pept. Sci. 60, 96–123 (2001). 10.1002/1097-0282(2001)60:2<96::AID-BIP1007>3.0.CO;2-F 11455545

[b47] Hornak V. . Comparison of multiple Amber force fields and development of improved protein backbone parameters. Proteins 65, 712–725 (2006). 16981200 10.1002/prot.21123 PMC4805110

[b48] Onufriev A., Bashford D. & Case D. A. Exploring protein native states and large-scale conformational changes with a modified generalized born model. Proteins 55, 383–394 (2004). 15048829 10.1002/prot.20033

