# Supplementary material for: Chiral Sulfoxide-Induced Single Turn Peptide α-Helicity
Source: Sci Rep. 2016 Dec 9;6:38573. doi: 10.1038/srep38573 (PMC5146914; doi:10.1038/srep38573)

Chiral Sulfoxide Induced Single Turn Peptide α-Helicity

Qingzhou Zhang[1], Fan Jiang[1], Bingchuan Zhao[1], Huacan Lin[1], Yuan Tian[1], Mingsheng Xie[1], Guoyun Bai[2], Adam M. Gilbert[2], Gilles H. Goetz[2], Spiros Liras[2], Alan A. Mathiowetz[2], David A. Price[2], Kun Song[2], Meihua Tu[2], Yujie Wu[4], Tao Wang[1,*], Mark E. Flanagan[3,*], Yun-Dong Wu[1,5,*] and Zigang Li[1,*]

1 School of Chemical Biology and Biotechnology, Peking University Shenzhen Graduate School, Shenzhen, 518055, China; E-mail: taowang@pkusz.edu.cn, wuyd@pkusz.edu.cn, lizg@pkusz.edu.cn

2 Cardiovascular and Metabolic Diseases Medicinal Chemistry, Pfizer, Inc., 620 Memorial Drive, Cambridge, MA, 02142, U. S. A.

3 Center for Chemistry Innovation and Excellence, Pfizer Inc., Eastern Point Road, Groton, CT, 06340, U.S.A.; E-mail: mark.e.flanagan@pfizer.com

4 Department of Biology, Southern University of Science and Technology, Shenzhen, China

5 College of Chemistry, Peking University, Beijing, 100871, China; E-mail: wuyd@pkusz.edu.cn

1. Abbreviations

2. General methods

3. Preparation and characterization of amino acid Xn and peptides

4. SI Figures and tables

5. Crystal data

6. References

1. Abbreviations

Fmoc, 9-fluorenylmethyloxycarbonyl; HCTU, 2-(1H-6-chlorobenzotriazol-1-yl)-1,1,3,3-tetramethyluronium hexafluorophosphate; DIPEA, diisopropylethylamine; DMF, dimethylformamide; ESI-MS, electrospray ionization mass spectrometry; MBHA, 4-methyl-benzylhydrylamine; RP-HPLC, reserved-phase high performance liquid chromatography; RT, room temperature; SPPS, solid-phase peptide synthesis; tBu, tert-butyl; TFA, trifluoroacetic acid; TFE, 2,2,2-trifluoroethanol; TIS, triisopropylsilane; Trt, triphenylmethyl; Et_2O, diethyl ether; EDT, 1, 2-ethanedithiol; LC-MS, liquid chromatography–mass spectrometry; NMR, Nuclear magnetic resonance; S-AA, stapling amino acid; HPLC, high-performance liquid chromatography; DMPA, 2, 2-dimethoxy-2-phenylacetophenone; S-BSN, (S)-(-)-2-Methyl-2-propanesulfinamide; R-BSN, (R)-(+)-2-Methyl-2-propanesulfinamide.

2. General methods

All reagents, amino acids and resins were purchased from GL Biochem (Shanghai), Shanghai Hanhong Chemical Co., J&K Scientific or Energy Chemical and were used without further purification. S-BSN and R-BSN were purchased from Meryerchemical. Unnatural amino acids are synthesized following literature[1]. NMP were purchased from Shenzhen Tenglong Logistics Co. and used without purification. All solvents used were bought from Cantotech Chemicals, Ltd.. DMF were distilled under reduced pressure from calcium hydride immediately prior to use. NMRs are measured on nuclear magnetic resonance (NMR) spectroscopy (Bruker AVANCE-III300, 400 and 500). 2D NMR were taken on Bruker AVANCE III 500 MHz spectrometer. Peptides were purified by HPLC (SHIMAZU Prominence LC-20AT or WATERS 600) using reverse phase C18 column Grace Vydac protein and peptide C18 250×22 mm (or Agilent Eclipse XDB-C18, 9.4×250 mm), flow rate 5mL/min or grace smart C18 250×10 mm flow rate 1 mL/min (or Anilent Poroshell 120 SB-C18 3.0×75 mm, flow rate 0.8 mL). Two kind of buffers for RP HPLC are buffer A (0.1% TFA in water) and buffer B (pure acetonitrile). LC-MS was measured on SHIMAZU-SPD2020; CD spectroscopys were measure on Chirascan Circular Dichroism Spectrometer.

General analytical method A: Peptides were eluted from a C18 (Anilent Poroshell 120 SB-C18 3.0×75 mm, flow rate 0.8 mL) using a gradient of 5% buffer B in buffer A to 50% buffer B over 7 min.

General analytical method B: Peptides were eluted from a C18 (Agilent Eclipse XDB-C18, 9.4×250 mm, flow rate 5.0 mL) using a gradient of 0% buffer B in buffer A over 11 min then to 25% buffer B over another 25 min.

3. Preparation of amino acids and peptides

Amino acids synthesis

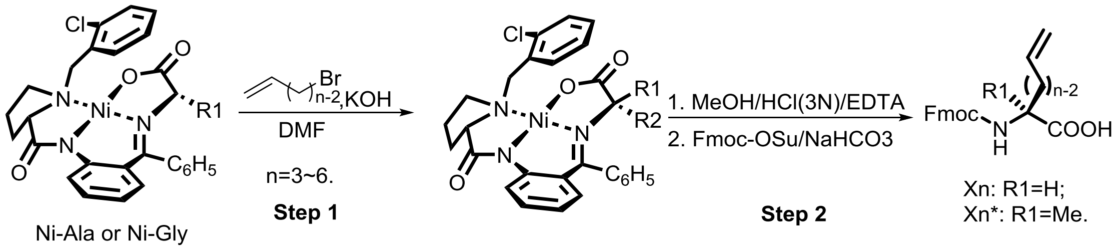

Step 1. To a stirred solution of **Ni-Gly** (10.6 g, 20 mmol) in DMF (200 mL) at 0 ℃ was added powdered KOH (11.2 g, 200 mmol, 10 equiv). The reaction mixture was stirred at 0 ℃ for 1 h, and then added 20 mmol (1.0 equiv) of allyl bromide (homoallyl bromide/4-pentenyl bromide/5-hexenyl bromide). The reaction mixture was warmed up to ambient temperature during 3 h, and then quenched slowly with 5% aqueous solution of AcOH to pH 5-7, stirred vigorously overnight. The mixture was filtered and washed with water for 3 time, then collected the red solid and dried under vacuum. The red solid was used in the step 2 without purification.

To a stirred solution of **Ni-Ala** (10.9 g, 20 mmol) in DMF (200 mL) at 0 ℃ was added 3 equiv of sodium hydride. The reaction mixture was stirred at 0 ℃ for 1 h, and then added 60 mmol (3.0 equiv) of allyl bromide (homoallyl bromide/4-pentenyl bromide/5-hexenyl bromide). The reaction mixture was warmed up to ambient temperature during 3 h, and then quenched slowly with 5% aqueous solution of AcOH to pH 5-7, stirred vigorously overnight. The mixture was filtered and washed with water for 3 time, then collected the red solid and dried under vacuum. The red solid was used in the step 2 without purification.

Step 2. The red solid from step 1 was dissolved in MeOH/HCl (3N) (100 mL, *v*/*v* = 1/1) cocktail, and then EDTA (5.8 g, 20 mmol) was added. The mixture was refluxed for 6 h. The reaction mixture was concentrated *in vacuo* till half of the volume remained. Then saturated $NaHCO_3$ was added with stirring till pH 7, and then a solution of Fmoc-OSu (5.1g, 15 mmol) in THF (70 mL) was added at ambient temperature. After for 3 h, the reaction was quenched with 1 N HCl, and extracted with ethyl acetate (200mL × 3), and the combined organic layer was dried over Na_2SO_4, filtered, and concentrated *in vacuo*. The residue was purified by flash column chromatography (silica gel, ethyl acetate/hexanes = 1/4). The yield of the products Xn and Xn* varied from 30% to 50%.

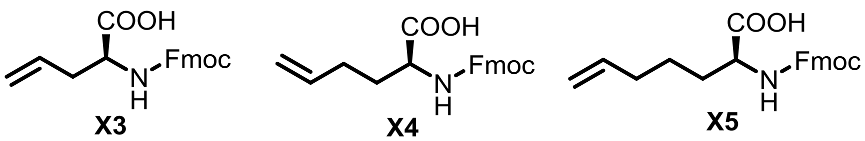

X3 **X4** **X5**

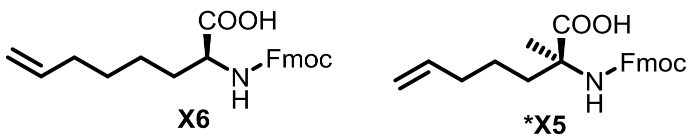

X6 ***X5**

The proton NMR of **X3**, **X4** and **X5** is published in literature[1].

X6 ^{1}H NMR (300 MHz, $CDCl_3$) δ 7.79 (d, *J* = 7.4 Hz, 2H), 7.62 (d, *J* = 6.6 Hz, 2H), 7.42 (t, *J* = 7.4 Hz, 2H), 7.33 (t, *J* = 7.3 Hz, 2H), 5.87 – 5.72 (m, 1H), 5.26 (d, *J* = 8.2 Hz, 1H), 5.01 (dd, *J* = 23.2, 6.0 Hz, 2H), 4.44 (d, *J* = 6.5 Hz, 3H), 4.25 (t, *J* = 6.8 Hz, 1H), 2.08 (d, *J* = 6.4 Hz, 2H), 2.01 – 1.88 (m, 1H), 1.75 (m, 1H), 1.44 (m, 4H).

***X5** ^{1}H NMR (300 MHz, $CDCl_3$) δ 7.78 (d, *J* = 7.4 Hz, 2H), 7.61 (d, *J* = 7.7 Hz, 2H), 7.41 (t, *J* = 7.1 Hz, 2H), 7.37 – 7.33 (t, *J* = 7.3 Hz, 2H), 5.85 – 5.69 (m, 1H), 5.57 (s, 1H), 4.99 (t, *J* = 12.9 Hz, 2H), 4.42 (t, *J* = 7.7 Hz, 2H), 4.23 (t, *J* = 6.4 Hz, 1H), 2.08 (m, 3H), 1.86 (m, 1H), 1.62 (s, 3H), 1.43 (m, 2H).

Peptide synthesis

General procedure A. Thioether linker is formed by intermolecular thiolene reaction, and then the linear peptide was cleaved from resin and cyclized in liquid phase.

Synthesis of Peptide 1 and 2

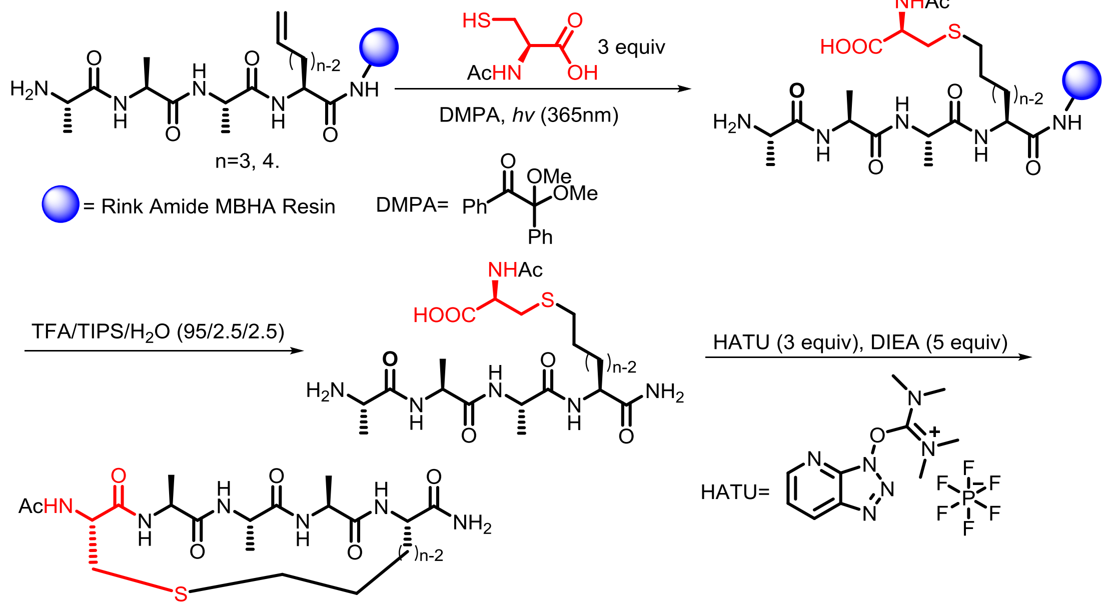

peptide 1, 2 (n= 3, 4).

1) H_2N-Ala-Ala-Ala-Xn-Resin (189 mg, 0.1 mmol) was prepared using Fmoc chemistry on Rink amide MBHA resin.
2) H_2N-Ala-Ala-Ala-Xn-Resin (0.1 mmol) was dissolved in anhydrous DMF (10 mL) at ambient temperature, and swelled for 20 min. N-Acetyl-L-cysteine (98mg, 0.3 mmol, 3.0 equiv) and DMAP (26 mg, 0.1 mmol, 1.0 equiv) was added, and the reaction was degassed, UV irradiated for 1 h with stirring. After photo reaction, the resin was washed with DCM for three times and then with methanol to shrink the resin. The resin was dried under a steam of argon gas for 1 hour.
3) The dried peptide-containing resin was placed in a polypropylene container with a screw cap, then cleavage cocktail (2.0 mL, TFA/TIS/EDT/H_2O 94/1/2.5/2.5) was added and the container was sealed tightly with screw cap. The container was gently agitated on an orbital shaker in the fume hood for 2 h. The TFA cocktail was removed with by evaporation under a steam of argon gas in the fume hood, and the residue was precipitated with cold diethyl ether 3×3 ml.
4) The precipitate was dried and dissolved in 100 mL dry DMF, and then HATU (115 mg, 0.3 mmol) and DIEA (82 uL, 0.5 mmol) were added at 0 °C. The mixture was stirred overnight and then concentrated *in vacuo*. The residue was dissolved in H_2O and purified on HPLC.

General procedure B. Thiolether linker was constructed through on-resin cyclization.

Synthesis of peptide 3 and 4

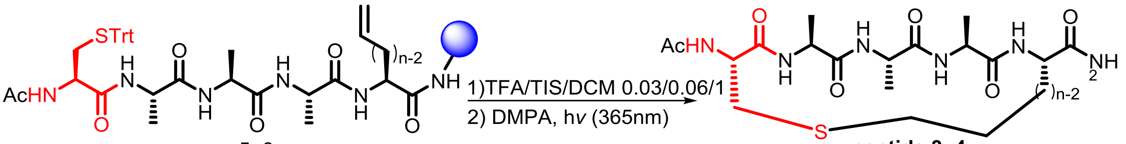

Ac-Cys(Trt)-Ala-Ala-Ala-Xn-Resin (189 mg, 0.1 mmol) was prepared using Fmoc chemistry on Rink amide MBHA resin.

1) Ac-Cys(Trt)-Ala-Ala-Ala-Xn-Resin (0.1mmol) was treated with 2 mL scavenger (TFA/TIS/DCM 0.03/0.06/1.0) for 1 h to remove Trt protection.
2) Ac-Cys-Ala-Ala-Ala-Xn-Resin was dried and re-dissolved in 5 mL DMF, and then DMPA (26 mg, 0.1 mmol) was added and stirred under UV irradiation of 0.5 – 1 h. The resin was washed with DMF and DCM for three times, and then shrink with methanol. The resin was dried with a steam of argon gas for 1 h.
3) The dried peptide-containing resin was placed in a polypropylene container with a screw cap, then cleavage cocktail (2.0 mL, TFA/TIS/EDT/H_2O 94/1/2.5/2.5) was added and the container was sealed tightly with screw cap. The container was gently agitated on an orbital shaker in the fume hood for 2 h. The TFA cocktail was removed by evaporation under a steam of argon gas in the fume hood, and the residue was precipitated with cold diethyl ether 3×3 ml. The residue was dissolved in H_2O/acetonitrile 1:1 and purified on HPLC or used directly in the oxidation procedure.

General procedure C. This procedure was the same with General procedure A except for that Rink amide resin and N-Acetyl-L-cysteine were change to CTC resin and L-Cysteinamide monohydrochloride.

Synthetic procedure of Peptide 5 and 6

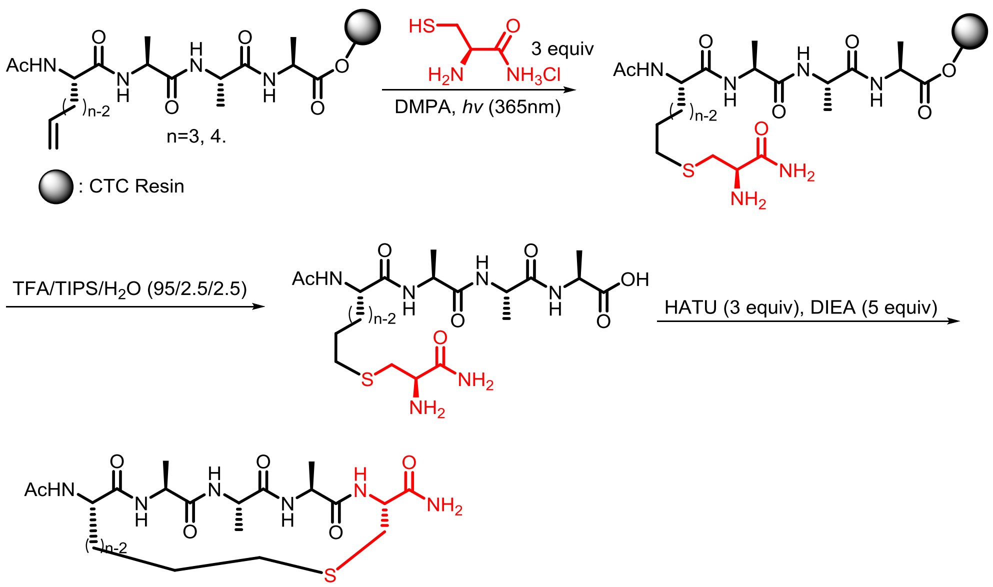

peptide 5, 6 (n= 3, 4).

Characterization of peptide 1-26

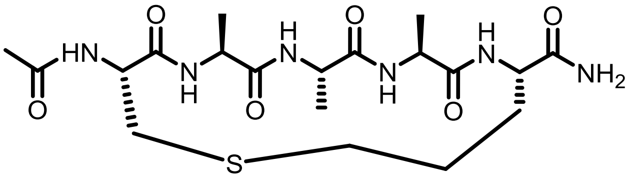

Chemical Formula: $C_{19}H_{32}N_6O_6S$
Exact Mass: 472.2104

Peptide **1** was synthesized following general procedure A. 15mg (32% isolated). General analytical method A, Rt: 2.68 min. MS $[M+H]^+$ = 473 (found), 473 (calc.). (^{1}H NMR, DMSO, 298K). δ 8.54 (d, J = 8.2 Hz, 1H, NH), 8.15 (d, J = 8.1 Hz, 1H, NH), 8.07 (d, J = 4.0 Hz, 1H, NH), 7.75 (d, J = 8.7 Hz, 1H, NH), 7.37 (d, J = 6.7 Hz, 1H, NH), 7.20 (s, 1H, C terminal NH), 7.00 (s, 1H, C terminal NH), 4.36 (d, J = 8.2 Hz, 1H, Hα), 4.22 – 4.11 (m, 3H, Hα), 4.01 (dd, J = 6.9, 4.1 Hz, 1H, Hα), 3.16 (dd, J = 13.0, 2.9 Hz, 1H, Cys Hβ), 2.65 – 2.54 (m, 2H, Cys Hβ, X3 Hδ), 2.33 (dd, J = 13.6, 8.6 Hz, 1H, X3 Hδ), 1.82 (s, 4H, Ac, X3 Hβ), 1.72 (d, J = 12.4 Hz, 1H, X3 Hβ), 1.61 – 1.49 (m, 2H, X3 Hγ), 1.27 – 1.14 (m, 9H, Ala2-4 Hβ X3 Hγ).

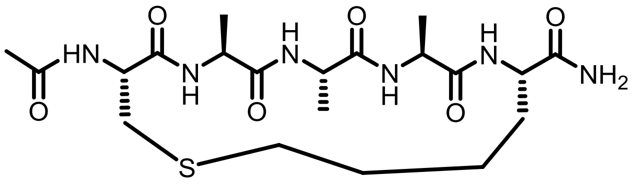

Chemical Formula: $C_{20}H_{34}N_6O_6S$
Exact Mass: 486.2261

Peptide **2** was synthesized following general procedure A. 11 mg (23% isolated). General analytical method A, Rt: 3.45 min. MS $[M+H]^+$ = 487 (found), 487 (calc.). (^{1}H NMR, DMSO, 298K). δ 8.61 (d, *J* = 7.7 Hz, 1H, NH), 8.12 (dd, *J* = 14.7, 6.4 Hz, 2H, NH), 7.74 (d, *J* = 7.9 Hz, 1H, NH), 7.41 (d, *J* = 6.5 Hz, 1H, NH), 7.20 (s, 1H, C terminal NH), 6.98 (s, 1H, C terminal NH), 4.41 (d, *J* = 5.4 Hz, 1H, Hα), 4.21 – 4.04 (m, 4H, Hα), 3.00 (dd, *J* = 13.7, 5.5 Hz, 1H, Cys Hβ), 2.64 – 2.54 (m, 3H, Cys Hβ, X4 Hβ), 1.83 (s, 3H, Ac), 1.61 (s, 3H, X4 Hβ Hβ), 1.46 (s, 2H, X4 Hγ δ), 1.22 (dd, *J* = 16.6, 6.3 Hz, 10H, Ala2-4 Hβ X4 Hδ).

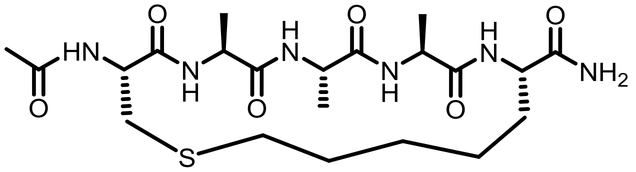

Chemical Formula: $C_{21}H_{36}N_6O_6S$
Exact Mass: 500.2417

Peptide **3** was synthesized following general procedure B. 9 mg (18% isolated). General analytical method A, Rt: 4.21 min. MS $[M+H]^+$ = 501 (found), 501 (calc.). (^{1}H NMR, DMSO, 298K). δ 8.60 (d, *J* = 7.9 Hz, 1H, NH), 8.18 (d, *J* = 7.7 Hz, 1H, NH), 8.04 (d, *J* = 6.1 Hz, 1H, NH), 7.76 (d, *J* = 8.9 Hz, 1H, NH), 7.29 (d, *J* = 6.7 Hz, 1H, NH), 7.12 (s, 1H, C terminal NH), 6.96 (s, 1H, C terminal NH), 4.38 (s, 1H, Hα), 4.26 – 4.06 (m, 4H, Hα), 2.82 (s, 2H, Cys Hβ), 2.60 (s, 2H, X5 Hζ), 1.82 (s, 3H, Ac), 1.71 – 1.59 (m, 3H, X5 Hε Hβ), 1.53 (s, 4H, X5 Hε Hδ Hγ), 1.22 (dd, *J* = 13.1, 6.3 Hz, 10H, Ala2-4 Hβ X5 Hδ).

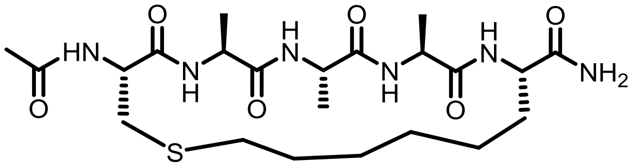

Chemical Formula: $C_{22}H_{38}N_6O_6S$
Exact Mass: 514.2574

Peptide **4** was synthesized following general procedure B. 16 mg (31% isolated). General analytical method A, Rt: 5.09 min. MS $[M+H]^+$ = 515 (found), 515 (calc.). (^{1}H NMR, DMSO, 298K). δ 8.57 (d, *J* = 7.2 Hz, 1H, NH), 8.16 (d, *J* = 7.6 Hz, 1H, NH), 8.01 (d, *J* = 7.0 Hz, 1H, NH), 7.70 (d, *J* = 7.8 Hz, 1H, NH), 7.31 (d, *J* = 7.2 Hz, 1H, NH),

7.11 (s, 1H, C terminal NH), 6.93 (s, 1H, C terminal NH), 4.42 – 4.37 (m, 1H, Hα), 4.27 – 4.09 (m, 4H, Hα), 2.85 – 2.82 (m, 1H, Cys Hβ), 2.64 (dd, *J* = 13.2, 8.2 Hz, 1H, Cys Hβ), 2.59 – 2.54 (m, 1H, X6 Hη), 2.43 (d, *J* = 8.6 Hz, 1H, X6 Hη), 1.84 (s, 3H, Ac), 1.58 (d, *J* = 55.8 Hz, 5H, X6 Hζ Hγ Hβ), 1.37 – 1.15 (m, 14H,Ala2-4 Hβ, X6 Hε Hδ Hγ).

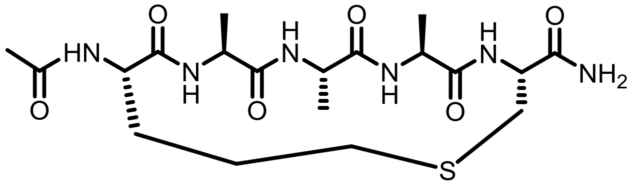

Chemical Formula: $C_{19}H_{32}N_6O_6S$
Exact Mass: 472.2104

Peptide **5** was synthesized following general procedure C. 11 mg (23% isolated). General analytical method A, Rt: 2.87 min. MS $[M+H]^+$ = 473 (found), 473 (calc.). (^{1}H NMR, DMSO, 298K). δ 8.34 (d, *J* = 7.7 Hz, 1H, NH), 8.09 (d, *J* = 5.6 Hz, 1H, NH), 8.03 (d, *J* = 7.7 Hz, 1H, NH), 7.88 (d, *J* = 8.3 Hz, 1H, NH), 7.74 (d, *J* = 6.2 Hz, 1H, NH), 7.18 (s, 1H, C terminal NH), 7.12 (s, 1H, C terminal NH), 4.27 (dd, *J* = 8.0, 4.3 Hz, 2H, Hα), 4.21 – 4.13 (m, 1H, Hα), 4.13 – 4.02 (m, 2H, Hα), 2.89 – 2.82 (m, 1H, Cys Hβ), 2.74 (dd, *J* = 13.9, 8.5 Hz, 1H, Cys Hβ), 2.54 (d, *J* = 3.6 Hz, 2H, X3 Hδ), 1.83 (s, 3H, Ac), 1.79 (s, 1H, X3 Hβ), 1.55 (s, 2H, X3 Hβ Hγ), 1.31 – 1.20 (m, 10H, Ala2-4 Hβ, X3 Hγ).

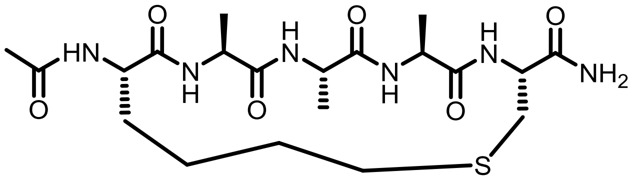

Chemical Formula: $C_{20}H_{34}N_6O_6S$
Exact Mass: 486.2261

Peptide **6** was synthesized following general procedure C. 7 mg (14% isolated). General analytical method A, Rt: 3.40 min. MS $[M+H]^+$ = 487 (found), 487 (calc.). (^{1}H NMR, DMSO, 298K). δ 8.61 (d, *J* = 7.7 Hz, 1H, NH), 8.12 (dd, *J* = 14.7, 6.4 Hz, 2H, NH), 7.74 (d, *J* = 7.9 Hz, 1H, NH), 7.41 (d, *J* = 6.5 Hz, 1H, NH), 7.20 (s, 1H, C terminal NH), 6.98 (s, 1H, C terminal NH), 4.41 (d, *J* = 5.4 Hz, 1H, Hα), 4.21 – 4.04 (m, 4H, Hα), 3.00 (dd, *J* = 13.7, 5.5 Hz, 1H, Cys Hβ), 2.64 – 2.54 (m, 3H, Cys Hβ, X4 Hε), 1.83 (s, 3H, Ac), 1.61 (s, 3H, X4 Hβ Hδ), 1.46 (s, 2H, X4 Hγ Hδ), 1.22 (dd, *J* = 16.6, 6.3 Hz, 10H, Ala2-4 Hβ, X4 Hδ).

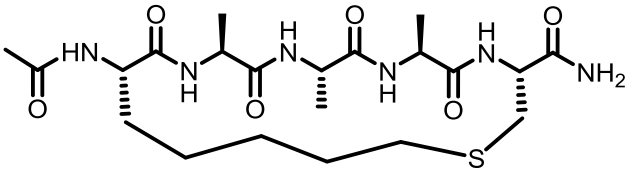

Chemical Formula: $C_{21}H_{36}N_6O_6S$
Exact Mass: 500.2417

Peptide **7** was synthesized following general procedure B. 10 mg (20% isolated). General analytical method A, Rt: 4.44 min. MS $[M+H]^+$ = 501 (found), 501 (calc.). (^{1}H NMR, DMSO, 298K). δ 8.38 (d, *J* = 7.3 Hz, 1H, NH), 8.20 (d, *J* = 5.9 Hz, 1H, NH), 8.00 (d, *J* = 7.8 Hz, 1H, NH), 7.95 (d, *J* = 8.1 Hz, 1H, NH), 7.39 (d, *J* = 6.4 Hz, 1H, NH), 7.21 (s, 1H, C terminal NH), 7.08 (s, 1H, C terminal NH), 4.29 – 4.05 (m, 5H, Hα), 2.84 (dd, *J* = 13.9, 4.4 Hz, 1H, Cys Hβ), 2.76 – 2.65 (m, 3H, Cys Hβ, X5 Hζ), 1.82 (s, 3H, Ac), 1.72 (s, 1H, X5 Hβ), 1.62 – 1.12 (m, 16H, Ala2-4 Hβ, X5 Hβ Hγ Hδ Hε Hζ).

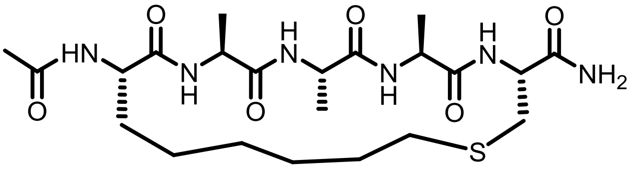

Chemical Formula: $C_{22}H_{38}N_6O_6S$
Exact Mass: 514.2574

Peptide **8** was synthesized following general procedure B. 14 mg (20% isolated). General analytical method A, Rt: 4.74 min. MS $[M+H]^+$ = 515 (found), 515 (calc.). **8** ^{1}H NMR (500 MHz, DMSO) δ 8.59 (d, *J* = 7.4 Hz, 1H, NH), 8.18 (d, *J* = 7.9 Hz, 1H, NH), 8.03 (d, *J* = 6.8 Hz, 1H, NH), 7.73 (d, *J* = 8.4 Hz, 1H, NH), 7.32 (d, *J* = 7.1 Hz, 1H, NH), 7.13 (s, 1H, C terminal NH), 6.95 (s, 1H, C terminal NH), 4.41 (d, *J* = 6.5 Hz, 1H, Hα), 4.27 – 4.09 (m, 4H, Hα), 2.84 (dd, *J* = 13.1, 6.1 Hz, 1H, Cys Hβ), 2.63 (dd, *J* = 13.2, 8.4 Hz, 1H, Cys Hβ), 2.57 (dd, *J* = 13.3, 5.5 Hz, 1H, X6 Hη), 2.45 – 2.35 (m, 2H, X6 Hη), 1.84 (s, 3H, Ac), 1.68 – 1.42 (m, 6H, X6 Hβ-ε), 1.41 – 1.11 (m, 13H, Ala2-4 Hβ, X6 Hβ-ε).

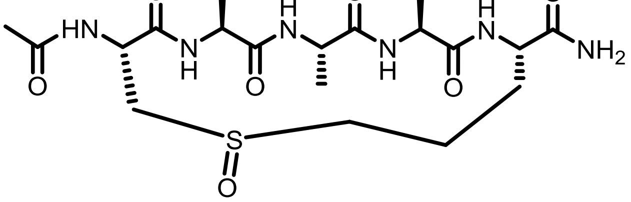

Chemical Formula: $C_{19}H_{32}N_6O_7S$
Exact Mass: 488.2053

Peptide **1** (10 mg) was dispersed in 5% H_2O_2 5 mL and the mixture was stirred for 3 h at ambient temperature, the result clear solution was purified on HPLC directly. **9AB** 8 mg (20% isolated). General analytical method A, Rt: 2.20/2.30 min. MS $[M+H]^+$ = 489 (found), 489 (calc.). (^{1}H NMR, DMSO, 298K). δ 8.65 (dd, *J* = 56.6, 8.1 Hz, 1H), 8.33 (dd, *J* = 19.7, 7.3 Hz, 1H), 8.11 (s, 1H), 7.91 (dd, *J* = 17.4, 8.5 Hz, 1H), 7.38 – 7.19 (m, 2H), 7.02 (d, *J* = 20.6 Hz, 1H), 4.76 – 4.59 (m, 1H), 4.24 – 4.12 (m, 4H), 3.95 (dd, *J* = 7.0, 3.6 Hz, 1H), 3.33 – 3.25 (m, 1H), 2.80 (dd, *J* = 17.2, 8.7 Hz, 2H), 2.67 (dd, *J* = 17.7, 8.5 Hz, 1H), 1.90 – 1.58 (m, 7H), 1.27 – 1.16 (m, 9H).

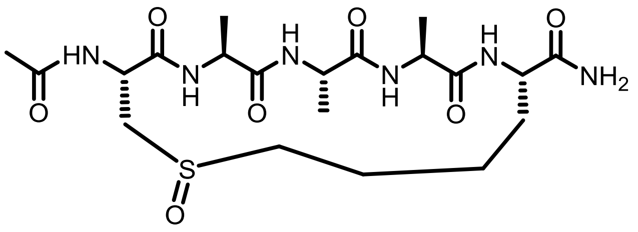

Chemical Formula: $C_{20}H_{34}N_6O_7S$
Exact Mass: 502.2210

Peptide **2** (10 mg) was dispersed in 5% H_2O_2 5 mL and the mixture was stirred for 3 h at ambient temperature, the result clear solution was purified on HPLC directly.

10A 3 mg (29% isolated). General analytical method A, Rt: 0.68 min. General analytical method B, Rt: 17.17 min. MS $[M+H]^+$ = 503 (found), 503 (calc.). ^{1}H NMR (300 MHz, DMSO, 298K) δ 8.85 (d, *J* = 8.3 Hz, 1H, NH), 8.37 (d, *J* = 7.8 Hz, 1H, NH), 8.13 (d, *J* = 6.2 Hz, 1H, NH), 7.63 (d, *J* = 8.7 Hz, 1H, NH), 7.46 (d, *J* = 7.3 Hz, 1H, NH), 7.32 (s, 1H, C terminal NH), 7.03 (s, 1H, C terminal NH), 4.71 (d, *J* = 4.4 Hz, 1H, Hα), 4.33 – 4.10 (m, 4H, Hα), 2.99 (d, *J* = 9.7 Hz, 1H, Cys Hβ), 2.88 (dd, *J* = 12.6, 5.4 Hz, 1H, Cys Hβ), 2.74 (d, *J* = 17.5 Hz, 1H, X4 Hε), 2.27 (d, *J* = 8.3 Hz, 1H, X4 Hε), 1.82 (s, 3H, Ac), 1.55 (s, 5H, X4 Hβ Hγ Hδ), 1.31 – 1.06 (m, 10H, Ala2-4 Hβ, X4 Hγ).

10B 4 mg (39% isolated). General analytical method A, Rt: 1.18 min. General analytical method B, Rt: 19.59 min. MS $[M+H]^+$ = 503 (found), 503 (calc.). ^{1}H NMR (300 MHz, DMSO, 298K) δ 8.62 (d, *J* = 7.5 Hz, 1H, NH), 8.34 (d, *J* = 8.0 Hz, 1H, NH), 8.06 (d, *J* = 5.0 Hz, 1H, NH), 7.84 (d, *J* = 8.7 Hz, 1H, NH), 7.40 – 7.24 (m, 2H, NH), 7.00 (s, 1H terminal NH), 4.64 (d, *J* = 5.5 Hz, 1H, Hα), 4.24 – 4.15 (m, 2H, Hα), 4.15 – 4.00 (m, 2H, Hα), 3.66 – 3.53 (m, 1H, Cys Hβ), 3.17 – 3.04 (m, 1H, Cys Hβ), 2.87 – 2.74 (m, 2H, X4 Hε), 1.82 (s, 3H, Ac), 1.62 (d, *J* = 6.5 Hz, 3H, X4 Hβ Hδ), 1.42 (d, *J* = 6.8 Hz, 2H, X4 Hγ Hδ), 1.22 (ddd, *J* = 16.6, 10.8, 6.1 Hz, 10H, Ala2-4 Hβ, X4 Hγ).

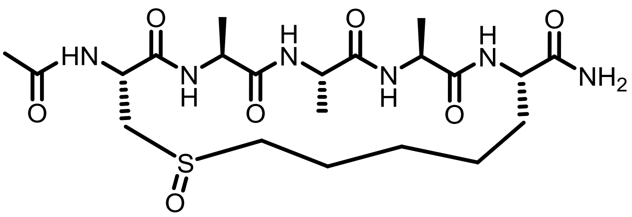

Chemical Formula: $C_{21}H_{36}N_6O_7S$
Exact Mass: 516.2366

Peptide **3** (10 mg) was dispersed in 5% H_2O_2 5 mL and the mixture was stirred for 3 h at ambient temperature, the result clear solution was purified on HPLC directly. **11AB** 7 mg (68% isolated). General analytical method A, Rt: 2.60/2.76 min. MS $[M+H]^+$ = 517 (found), 517 (calc.). ^1H NMR (500 MHz, DMSO, 298K) δ 8.65 (dd, *J* = 40.7, 7.8 Hz, 1H), 8.35 (dd, *J* = 19.6, 7.9 Hz, 1H), 8.10 (dd, *J* = 21.0, 6.2 Hz, 1H), 7.81 (d, *J* = 8.8 Hz, 1H), 7.27 (d, *J* = 7.9 Hz, 1H), 7.23 – 7.18 (m, 1H), 7.02 – 6.89 (m, 1H), 4.69 – 4.61 (m, 1H), 4.38 – 4.24 (m, 1H), 4.24 – 4.06 (m, 3H), 3.20 (ddd, *J* = 19.5, 13.4, 5.1 Hz, 1H), 2.96 – 2.84 (m, 2H), 2.81 – 2.68 (m, 1H), 1.84 (d, *J* = 9.2 Hz, 3H), 1.66 (d, *J* = 20.5 Hz, 3H), 1.28 (dddd, *J* = 21.2, 13.1, 10.6, 7.3 Hz, 14H).

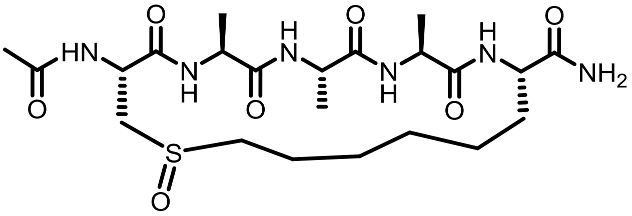

Chemical Formula: $C_{22}H_{38}N_6O_7S$
Exact Mass: 530.2523

Peptide **4** (10 mg) was dispersed in 5% H_2O_2 5 mL and the mixture was stirred for 3 h at ambient temperature, the result clear solution was purified on HPLC directly. **12AB** 9 mg (87% isolated). General analytical method A, Rt: 3.29/3.36 min. ^1H NMR (500 MHz, DMSO) δ 8.78 (s, 1H), 8.57 (d, *J* = 7.0 Hz, 1H), 8.37 – 8.29 (m, 1H), 8.08 (d, *J* = 6.9 Hz, 1H), 7.83 – 7.72 (m, 1H), 7.32 (dd, *J* = 17.8, 7.6 Hz, 1H), 7.18 - 6.90 (m, 1H), 4.68 (s, 1H), 4.27 (d, *J* = 7.2 Hz, 1H), 4.19 (dd, *J* = 15.7, 8.1 Hz, 2H), 4.11 – 4.01 (m, 1H), 3.01 (s, 1H), 2.87 – 2.77 (m, 1H), 1.83 (s, 3H), 1.64 (s, 1H), 1.54 – 1.46 (m, 1H), 1.33 (s, 3H), 1.30 – 1.10 (m, 10H).

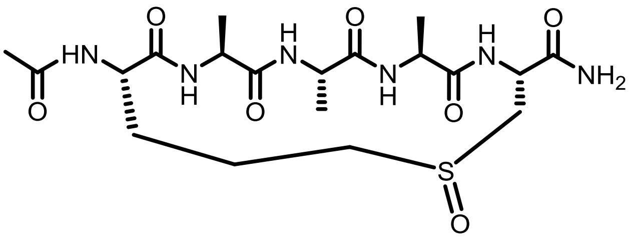

Chemical Formula: $C_{19}H_{32}N_6O_7S$
Exact Mass: 488.2053

Peptide **5** (12 mg) was dispersed in 5% H_2O_2 6 mL and the mixture was stirred for 3 h at ambient temperature, the result clear solution was purified on HPLC directly.

13A 4 mg (32% isolated). General analytical method A, Rt: 0.98 min. MS $[M+Na]^+$ = 511 (found), 511 (calc.). ^1H NMR (500 MHz, DMSO, 298K) δ 8.41 (s, 1H, NH), 8.19 (s, 1H, NH), 8.07 (dd, *J* = 10.2, 6.1 Hz, 2H, NH), 7.57 – 7.51 (m, 1H, NH), 7.20 (d, *J* = 22.9 Hz, 2H, C terminal NH_2), 4.56 (dd, *J* = 8.3, 4.2 Hz, 1H, Hα), 4.31 (d, *J* = 7.2 Hz, 1H, Hα), 4.24 – 4.18 (m, 1H, Hα), 4.12 – 4.07 (m, 1H, Hα), 3.98 – 3.90 (m, 1H, Hα), 3.16 – 3.11 (m, 1H, Cys Hβ), 3.07 – 3.04 (m, 1H, Cys Hβ), 2.82 – 2.77 (m, 1H, X3 Hδ), 2.74 – 2.68 (m, 1H, X3 Hδ), 1.91 (s, 1H, X3 Hβ), 1.84 (s, 3H, Ac), 1.74 – 1.60 (m, 3H, X3 Hβ Hγ), 1.32 – 1.21 (m, 9H, Ala2-4 Hβ).

13B 4 mg (32% isolated). General analytical method A, Rt: 1.41 min. MS $[M+Na]^+$ = 511 (found), 511 (calc.). ^1H NMR (500 MHz, DMSO) δ 8.45 (d, *J* = 7.5 Hz, 1H, NH), 8.11 (d, *J* = 4.6 Hz, 1H, NH), 8.05 (d, *J* = 8.0 Hz, 2H, NH), 7.57 (d, *J* = 5.7 Hz, 1H, NH), 7.34 (s, 1H, C terminal NH), 7.25 (s, 1H, C terminal NH), 4.51 (dd, *J* = 14.2, 5.5 Hz, 1H, Hα), 4.34 – 4.25 (m, 1H, Hα), 4.19 (t, *J* = 7.4 Hz, 1H, Hα), 4.14 – 4.03 (m, 1H, Hα), 3.96 (dd, *J* = 7.0, 4.9 Hz, 1H, Hα), 3.10 – 3.02 (m, 1H, Cys Hβ), 2.93 (dd, *J* = 13.2, 2.3 Hz, 1H, Cys Hβ), 2.87 – 2.76 (m, 2H, X3 Hδ), 1.95 (dd, *J* = 14.5, 6.3 Hz, 1H, X3 Hβ), 1.83 (s, 3H, Ac), 1.71 – 1.57 (m, 3H, X3 Hβ Hγ), 1.28 – 1.21 (m, 9H, Ala2-4 Hβ).

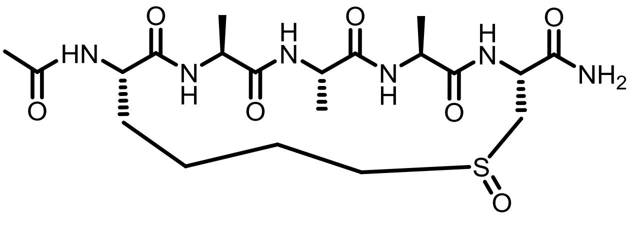

Chemical Formula: $C_{20}H_{34}N_6O_7S$
Exact Mass: 502.2210

Peptide **6** (5 mg) was dispersed in 5% H_2O_2 3 mL and the mixture was stirred for 3 h at ambient temperature, the result clear solution was purified on HPLC directly.

14A 2 mg (39% isolated). General analytical method A, Rt: 0.98 min. MS $[M+H]^+$ = 503 (found), 503 (calc.). ^1H NMR (500 MHz, DMSO, 298K) δ 8.42 (d, *J* = 7.5 Hz, 1H, NH), 8.27 (d, *J* = 7.9 Hz, 1H, NH), 8.21 (d, *J* = 4.6 Hz, 1H, NH), 8.00 (d, *J* = 8.0 Hz, 1H, NH), 7.30 (s, 1H, C terminal NH), 7.27 – 7.18 (m, 2H, NH), 4.64 (td, *J* = 7.5, 3.3 Hz, 1H, Hα), 4.34 – 4.26 (m, 1H, Hα), 4.22 (t, *J* = 6.9 Hz, 1H, Hα), 4.15 (t, *J* = 7.4 Hz, 1H, Hα), 4.00 (dd, *J* = 7.0, 4.7 Hz, 1H, Hα), 3.24 (dd, *J* = 14.0, 3.3 Hz, 1H, Cys Hβ), 2.99 (dd, *J* = 14.0, 7.3 Hz, 1H, Cys Hβ), 2.87 (dd, *J* = 13.1, 9.2 Hz, 1H, X4 Hε), 2.78 (dd, *J* = 16.7, 6.5 Hz, 1H, X4 Hε), 1.86 – 1.76 (m, 4H, Ac, X4 Hβ), 1.71 (s, 1H, X4 Hβ), 1.51 (d, *J* = 7.7 Hz, 3H, X4 Hδ Hγ), 1.35 (d, *J* = 7.1 Hz, 1H, X4 Hγ), 1.30 – 1.11 (m, 9H, Ala2-4 Hβ).

14B 2 mg (39% isolated). General analytical method A, Rt: 1.32 min. MS $[M+Na]^+$ = 525 (found), 525 (calc.). ^1H NMR (500 MHz, DMSO, 298K) δ 8.32 (d, *J* = 7.0 Hz, 1H, NH), 8.17 (dd, *J* = 22.7, 6.7 Hz, 2H, NH), 7.97 (d, *J* = 7.8 Hz, 1H, NH), 7.48 – 7.34 (m, 2H, NH), 7.21 (s, 1H, C terminal NH), 4.52 (dd, *J* = 14.0, 5.4 Hz, 1H, Hα), 4.31 (dd, *J* = 12.4, 7.2 Hz, 1H, Hα), 4.16 (dd, *J* = 13.2, 6.6 Hz, 2H, Hα), 4.05 – 3.93 (m, 1H, Hα), 3.15 – 3.05 (m, 1H, Cys Hβ), 3.05 – 2.95 (m, 2H, Cys Hβ, X4 Hε), 2.74 (dd, *J* = 11.9, 6.8 Hz, 1H, X4 Hε), 1.82 (s, 3H, Ac), 1.78 – 1.65 (m, 2H, X4 Hβ), 1.65 – 1.49 (m, 3H, X4 Hγ Hδ), 1.45 (d, *J* = 8.8 Hz, 1H, X4 Hδ), 1.29 – 1.10 (m, 9H, Ala2-4 Hβ).

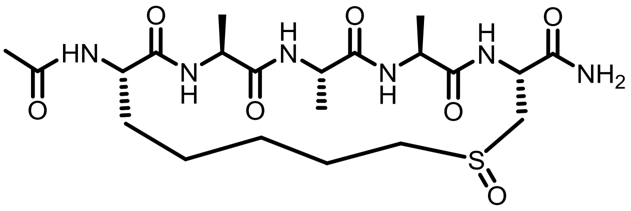

Chemical Formula: $C_{21}H_{36}N_6O_7S$
Exact Mass: 516.2366

Peptide **7** (15 mg) was dispersed in 5% H_2O_2 7 mL and the mixture was stirred for 3 h at ambient temperature, the result clear solution was purified on HPLC directly.

15A 5 mg (32% isolated). General analytical method A, Rt: 2.36 min. General analytical method B, Rt: 22.65 min. MS $[M+H]^+$ = 517 (found), 517 (calc.). ^1H NMR (500 MHz, DMSO, 298K) δ 8.36 (d, *J* = 7.6 Hz, 1H, NH), 8.22 (d, *J* = 7.6 Hz, 2H, NH), 7.95 (d, *J* = 7.9 Hz, 1H, NH), 7.54 (s, 1H, C terminal NH), 7.19 (d, *J* = 7.6 Hz, 2H, NH), 4.46 (d, *J* = 6.2 Hz, 1H, Hα), 4.26 – 4.17 (m, 2H, Hα), 4.17 – 4.10 (m, 1H, Hα), 4.03 – 3.96 (m, 1H, Hα), 3.14 – 3.04 (m, 2H, Cys Hβ), 2.84 (s, 1H, X5 Hζ), 2.67 (d, *J* = 9.9 Hz, 1H, X5 Hζ), 1.81 (s, 3H, Ac), 1.68 (s, 2H, X5 Hβ), 1.54 (s, 1H, X5 Hγ), 1.36 (s, 4H, X5 Hγ Hδ Hε), 1.29 – 1.16 (m, 10H, Ala2-4 Hβ, X5 Hδ).

15B 8 mg (52% isolated). General analytical method A, Rt: 3.54 min. General analytical method B, Rt: 27.40 min. MS $[M+H]^+$ = 517 (found), 517 (calc.). ^1H NMR (500 MHz,

DMSO, 298K) δ 8.42 (d, *J* = 6.9 Hz, 1H, NH), 8.12 (d, *J* = 6.2 Hz, 1H, NH), 8.07 (d, *J* = 5.7 Hz, 1H, NH), 7.95 (d, *J* = 8.7 Hz, 1H, NH), 7.28 (d, *J* = 10.4 Hz, 2H, NH), 7.20 (s, 1H, C terminal NH), 4.57 (t, *J* = 8.4 Hz, 1H, Hα), 4.13 (dd, *J* = 14.5, 7.4 Hz, 3H, Hα), 4.06 – 3.97 (m, 1H, Hα), 3.08 – 2.94 (m, 1H, Cys Hβ), 2.87 (dd, *J* = 13.2, 7.1 Hz, 2H, Cys Hβ, X5 Hζ), 2.70 – 2.60 (m, 1H, X5 Hζ), 1.87 (s, 3H, Ac), 1.79 (dd, *J* = 18.3, 11.6 Hz, 2H, X5 Hβ), 1.60 – 1.47 (m, 3H, X5 Hγ Hε), 1.32 (s, 3H, X5 Hγ Hδ), 1.24 (t, *J* = 6.5 Hz, 10H, Ala2-4 Hβ, X5 Hδ).

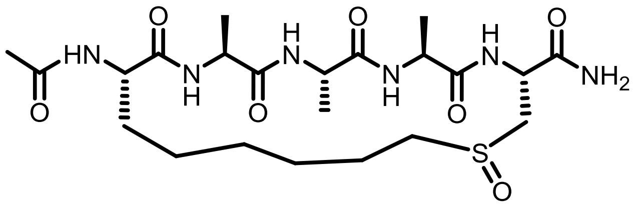

Chemical Formula: $C_{22}H_{38}N_6O_7S$
Exact Mass: 530.2523

Peptide **8** (10 mg) was dispersed in 5% H_2O_2 5 mL and the mixture was stirred for 3 h at ambient temperature, the result clear solution was purified on HPLC directly.

16A 4 mg (39% isolated). General analytical method A, Rt: 3.50 min. MS $[M+H]^+$ = 531 (found), 531 (calc.). ^{1}H NMR (500 MHz, DMSO, 298K) δ 8.42 (d, *J* = 7.6 Hz, 1H, NH), 8.33 (d, *J* = 8.2 Hz, 1H, NH), 8.19 (d, *J* = 6.4 Hz, 1H, NH), 7.99 (d, *J* = 7.9 Hz, 1H, NH), 7.24 – 7.16 (m, 3H, NH), 4.68 – 4.61 (m, 1H, Hα), 4.30 – 4.08 (m, 3H, Hα), 3.17 (dd, *J* = 13.8, 3.0 Hz, 1H, Cys Hβ), 3.02 (dd, *J* = 13.9, 9.3 Hz, 1H, Cys Hβ), 2.71 (dd, *J* = 13.7, 6.5 Hz, 2H, X6 Hη), 1.81 (s, 3H, Ac), 1.71 (m, 1H, X6 Hβ), 1.59 (dt, *J* = 24.3, 7.7 Hz, 2H, X6 Hβ Hζ), 1.52 – 1.40 (m, 3H, X6 Hγ Hζ), 1.40 – 1.18 (m, 13H, Ala2-4 Hβ, X6 Hδ Hε).

16B 4 mg (39% isolated). General analytical method A, Rt: 3.64 min. MS $[M+H]^+$ = 531 (found), 531 (calc.). ^{1}H NMR (500 MHz, DMSO, 298K) δ 8.41 (d, *J* = 7.4 Hz, 1H, NH), 8.20 (d, *J* = 6.8 Hz, 1H, NH), 8.13 (d, *J* = 8.7 Hz, 1H, NH), 7.98 (d, *J* = 7.7 Hz, 1H, NH), 7.39 (s, 1H, C terminal NH), 7.20 (d, *J* = 7.5 Hz, 2H, NH), 4.54 (t, *J* = 9.7 Hz, 1H, Hα), 4.29 – 4.13 (m, 4H, Hα), 3.08 – 2.93 (m, 2H, Cys Hβ), 2.74 (td, *J* = 13.3, 6.4 Hz, 2H, X6 Hη), 1.82 (s, 3H, Ac), 1.64 (t, *J* = 18.4 Hz, 3H, X6 Hβ Hζ), 1.44 (d, *J* = 6.9 Hz, 3H, X6 Hγ Hζ), 1.38 – 1.17 (m, 13H, Ala2-4 Hβ, X6 Hδ Hε).

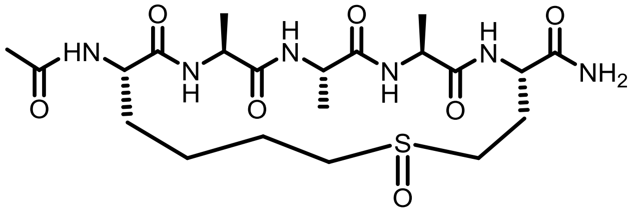

Chemical Formula: $C_{21}H_{36}N_6O_7S$
Exact Mass: 516.2366

The thiolether peptide Ac-*c*(1,5)-[X4AAAC*]-Resin (0.1 mmol) was synthesized following general procedure B. After cleavage, the precipitate was dissolved in 5% H_2O_2 (5 mL) and the mixture was stirred at room temperature for 3 h. Then the solution was purified on RP HPLC.

17A 3 mg (6% isolated based on resin loading). General analytical method A, Rt: 1.75 min. General analytical method B, Rt: 24.40 min. MS $[M+H]^+$ = 517 (found), 517 (calc.). ^1H NMR (500 MHz, DMSO, 298K) δ 8.43 (d, *J* = 7.8 Hz, 1H, NH), 8.15 (d, *J* = 5.7 Hz, 1H, NH), 8.07 (d, *J* = 8.5 Hz, 1H, NH), 8.01 (d, *J* = 7.9 Hz, 1H, NH), 7.32 (s, 1H, C terminal NH), 7.11 – 7.02 (m, 2H, NH), 4.29 (dd, *J* = 16.1, 9.1 Hz, 2H, Hα), 4.21 (m, 2H, Hα), 4.06 (m, 1H, Hα), 3.47 – 3.40 (m, 2H, Cys* Hγ), 2.79 (m, 1H, X4 Hε), 2.07 (m, 1H, X4 Hε), 1.82 (m, 4H, Ac, Cys* Hβ), 1.69 – 1.58 (m, 3H, Cys* Hβ, X4 Hβ), 1.48 (m, 4H, X4 Hγ Hδ), 1.28 – 1.16 (m, 9H, Ala2-4 Hβ).

17B 4 mg (8% isolated). General analytical method A, Rt: 2.94 min. General analytical method B, Rt: 25.91 min. MS $[M+H]^+$ = 517 (found), 517 (calc.). ^1H NMR (500 MHz, DMSO, 298K) δ 8.45 (d, *J* = 8.1 Hz, 1H, NH), 8.12 (d, *J* = 5.4 Hz, 1H, NH), 8.07 (d, *J* = 8.1 Hz, 1H, NH), 7.99 (d, *J* = 8.9 Hz, 1H, NH), 7.31 (s, 1H, C terminal NH), 7.09 (d, *J* = 7.1 Hz, 2H, NH), 4.34 (s, 1H, Hα), 4.31 – 4.22 (m, 2H, Hα), 4.22 – 4.16 (m, 1H, Hα), 4.08 (d, *J* = 6.7 Hz, 1H, Hα), 3.42 (m, 1H, Cys* Hγ), 2.84 (m, 1H, Cys* Hγ), 2.73 (d, *J* = 9.9 Hz, 2H, X4 Hε), 2.18 (m, 1H, Cys* Hβ), 1.79 (m, 6H, Ac, Cys* Hβ, X4 Hβ), 1.57 (m, 4H, X4 Hγ Hδ), 1.34 – 1.16 (m, 9H, Ala2-4 Hβ).

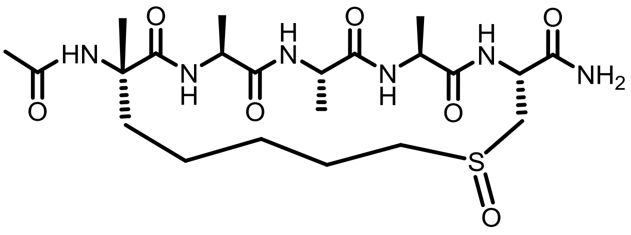

Chemical Formula: $C_{22}H_{38}N_6O_7S$
Exact Mass: 530.2523

The thiolether peptide Ac-*c*(1,5)-[X5*AAAC]-Resin (0.1 mmol) was synthesized following general procedure B. After cleavage, the precipitate was dissolved in 5% H_2O_2

(5 mL) and the mixture was stirred at room temperature for 3 h. Then the solution was purified on RP HPLC.

18A 2 mg (4% isolated based on resin loading). General analytical method A, Rt: 2.94 min. General analytical method B, Rt: 29.55 min. MS $[M+H]^+$ = 531 (found), 531 (calc.). ^{1}H NMR (500 MHz, DMSO, 298K) δ 8.14 (s, 1H, NH), 8.00 (s, 2H, NH), 7.65 (d, *J* = 25.9 Hz, 2H, NH), 7.24 (s, 2H, NH), 4.53 (s, 1H, Hα), 4.19 – 4.01 (m, 2H, Hα), 3.13 (d, *J* = 8.1 Hz, 1H, Cys Hβ), 2.97 (s, 3H, Cys Hβ, X5* Hζ), 1.90 (s, 1H, X5* Hβ), 1.84 (s, 3H, Ac), 1.69 (s, 2H, X5* Hβ Hε), 1.59 (s, 1H, X5* Hε), 1.40 (s, 3H, S5* Hβ(α–Methyl)), 1.33 – 1.19 (m, 13H, Ala2-4 Hβ, X5* Hγ Hδ).

18B 2 mg (4% isolated based on resin loading). General analytical method A, Rt: 4.05 min. General analytical method B, Rt: 31.14 min. MS $[M+H]^+$ = 531 (found), 531 (calc.). ^{1}H NMR (500 MHz, DMSO, 298K) δ 8.38 (s, 1H, NH), 8.08 (s, 1H, NH), 7.76 – 7.63 (m, 3H, NH), 7.37 (s, 1H, NH), 7.07 (s, 1H, NH), 4.46 (s, 1H, Hα), 4.21 – 4.13 (m, 1H, Hα), 4.04 – 3.94 (m, 2H, Hα), 3.04 (d, *J* = 11.1 Hz, 1H, Cys Hβ), 2.84 (s, 1H, Cys Hβ), 2.64 (s, 2H, X5* Hζ), 1.88 (s, 3H, Ac), 1.69 (s, 2H, X5* Hβ), 1.54 (s, 2H, X5* Hε), 1.39 (s, 3H, S5* Hβ(α–Methyl)), 1.32 – 1.18 (m, 13H, Ala2-4 Hβ, X5* Hγ Hδ).

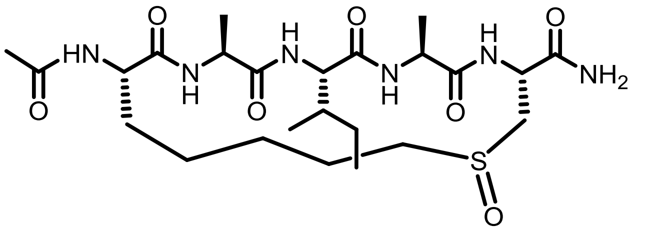

Chemical Formula: $C_{24}H_{42}N_6O_7S$
Exact Mass: 558.2836

The thiolether peptide Ac-*c*(1,5)-[X5AIAC]-NH_2 (0.3 mmol) was synthesized following general procedure C. After remove DMF, the precipitate was dissolved in 5% H_2O_2 (5 mL) and the mixture was stirred at room temperature for 3 h. Then the solution was purified on RP HPLC.

19A 7 mg (4% isolated based on resin loading). General analytical method A, Rt: 3.99 min. General analytical method B, Rt: 29.41 min. MS $[M+H]^+$ = 559 (found), 559 (calc.). ^{1}H NMR (500 MHz, DMSO, 298K) δ 8.48 (d, *J* = 7.8 Hz, 1H, NH), 8.30 (d, *J* = 7.9 Hz, 2H, NH), 8.01 (d, *J* = 7.7 Hz, 1H, NH), 7.22 (s, 1H, NH), 7.13 (s, 1H, NH), 6.53 (s, 1H, NH), 4.49 (s, 1H, Hα), 4.23 (dd, *J* = 18.5, 7.6 Hz, 2H, Hα), 4.13 (d, *J* = 5.8 Hz, 1H, Hα), 3.99 – 3.93 (m, 1H, Hα), 3.09 (d, *J* = 6.3 Hz, 2H, Cys Hβ), 2.80 (s, 1H, X5 Hζ), 2.68 – 2.63 (m, 1H, X5 Hζ), 1.81 (s, 3H, Ac), 1.71 (s, 3H, X5 Hβ Hε), 1.55 (s, 1H), 1.37 (s, 6H), 1.27 – 1.12 (m, 6H), 1.07 (s, 2H), 0.88 – 0.75 (m, 5H).

19B 20 mg (12% isolated based on resin loading). General analytical method A, Rt: 4.95 min. General analytical method B, Rt: 35.57 min. MS $[M+H]^+$ = 559 (found), 559 (calc.). ^1H NMR (500 MHz, DMSO, 298K) δ 8.53 (d, *J* = 7.4 Hz, 1H, NH), 8.17 (d, *J* = 6.8 Hz, 1H, NH), 8.11 (d, *J* = 5.1 Hz, 1H, NH), 8.02 (d, *J* = 8.8 Hz, 1H, NH), 7.26 (s, 1H, NH), 7.21 (s, 1H, NH), 6.90 (d, *J* = 8.6 Hz, 1H, NH), 4.62 (d, *J* = 8.5 Hz, 1H, Hα), 4.16 – 4.05 (m, 3H, Hα), 4.01 – 3.94 (m, 1H, Hα), 3.10 – 3.02 (m, 1H, Cys Hβ), 3.02 – 2.88 (m, 2H, Cys Hβ, X5 Hζ), 2.61 (s, 1H, X5 Hζ), 1.85 (s, 3H, Ac), 1.74 (d, *J* = 38.5 Hz, 3H, X5 Hβ Hε), 1.56 (s, 5H), 1.33 (s, 3H), 1.23 (dd, *J* = 18.2, 7.2 Hz, 6H), 1.05 (s, 1H), 0.89 – 0.73 (m, 5H).

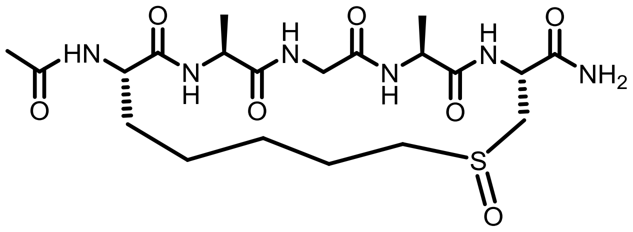

Chemical Formula: $C_{20}H_{34}N_6O_7S$
Exact Mass: 502.2210

The thiolether peptide Ac-*c*(1,5)-[X5AGAC]-Resin (0.1 mmol) was synthesized following general procedure B. After cleavage, the precipitate was dissolved in 5% H_2O_2 (5 mL) and the mixture was stirred at room temperature for 3 h. Then the solution was purified on RP HPLC.

20A 4 mg (8% isolated based on resin loading). General analytical method A, Rt: 2.64 min. General analytical method B, Rt: 21.09 min. MS $[M+H]^+$ = 503 (found), 503 (calc.). ^1H NMR (500 MHz, DMSO, 298K) δ 8.33 (dd, *J* = 11.3, 8.3 Hz, 2H, NH), 8.17 (d, *J* = 6.3 Hz, 1H, NH), 7.94 (d, *J* = 7.5 Hz, 1H, NH), 7.46 (s, 1H, NH), 7.31 (s, 1H, NH), 7.22 (s, 1H, NH), 4.60 (d, *J* = 5.2 Hz, 1H, Hα), 4.36 – 4.28 (m, 1H, Hα), 4.25 (d, *J* = 7.3 Hz, 1H, Hα), 4.17 – 4.09 (m, 1H, Hα), 3.81 (dd, *J* = 17.1, 5.2 Hz, 1H), 3.66 (dd, *J* = 17.1, 4.7 Hz, 1H, Hα), 3.15 (dd, *J* = 13.4, 5.0 Hz, 1H, Cys Hβ), 2.95 (dd, *J* = 13.5, 7.8 Hz, 1H, Cys Hβ), 2.82 – 2.72 (m, 1H, X5 Hζ), 2.66 (dd, *J* = 14.1, 8.1 Hz, 1H, X5 Hζ), 1.82 (s, 3H, Ac), 1.67 (s, 1H, X5 Hβ), 1.57 (d, *J* = 6.4 Hz, 2H, X5 Hβ Hε), 1.48 (s, 1H, X5 Hε), 1.43 – 1.18 (m, 10H, Ala2/Ala4 Hβ, X5 Hγ Hδ).

20B 5 mg (10% isolated based on resin loading). General analytical method A, Rt: 2.70 min. General analytical method B, Rt: 23.52 min. MS $[M+H]^+$ = 503 (found), 503 (calc.). ^1H NMR (500 MHz, DMSO, 298K) δ 8.37 (d, *J* = 7.7 Hz, 1H, NH), 8.19 (d, *J* = 6.7 Hz, 1H, NH), 8.10 (d, *J* = 8.8 Hz, 1H, NH), 7.94 (d, *J* = 7.6 Hz, 1H, NH), 7.40 (dd, *J* = 20.1, 15.0 Hz, 2H, NH), 7.24 (s, 1H, NH), 4.62 (t, *J* = 8.3 Hz, 1H, Hα), 4.30 – 4.12 (m, 3H, Hα), 3.72 (ddd, *J* = 34.5, 17.3, 5.2 Hz, 2H, Hα), 3.05 (s, 1H, Cys Hβ), 2.94 (s, 1H, Cys Hβ), 2.85 – 2.78 (m, 1H, X5 Hζ), 2.66 (d, *J* = 17.2 Hz, 1H, X5 Hζ), 1.83 (s, 3H, Ac), 1.73 (d, *J* = 5.8 Hz, 1H, X5 Hβ), 1.65 (d, *J* = 7.2 Hz, 1H, X5 Hβ), 1.58 (d, *J* = 7.5 Hz,

1H Hε), 1.47 (dd, J = 14.2, 6.2 Hz, 2H, X5 Hγ Hε), 1.40 – 1.16 (m, 9H, Ala2/Ala4 Hβ, X5 Hγ Hδ).

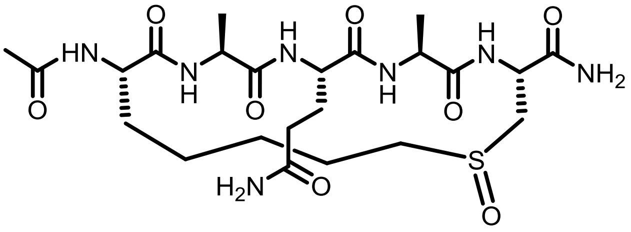

Chemical Formula: $C_{23}H_{39}N_7O_8S$
Exact Mass: 573.2581

The thiolether peptide Ac-*c*(1,5)-[X5AQAC]-Resin (0.1 mmol) was synthesized following general procedure B. After cleavage, the precipitate was dissolved in 5% H_2O_2 (5 mL) and the mixture was stirred at room temperature for 3 h. Then the solution was purified on RP HPLC.

21A 2 mg (4% isolated based on resin loading). General analytical method A, Rt: 2.13 min. General analytical method B, Rt: 21.72 min. MS $[M+H]^+$ = 574 (found), 574 (calc.). ^{1}H NMR (500 MHz, DMSO, 298K) δ 8.38 (s, 1H, NH), 8.32 (s, 2H, NH), 7.99 (s, 1H, NH), 7.37 (s, 1H, NH), 7.21 (s, 3H, NH), 6.77 (s, 1H, NH), 4.52 (s, 1H, Hα), 4.23 (s, 3H, Hα), 4.02 (s, 1H, Hα), 3.14 (s, 1H, Cys Hβ), 3.08 (s, 1H, Cys Hβ), 2.85 – 2.79 (m, 1H, X5 Hζ), 2.74 – 2.69 (m, 1H, X5 Hζ), 2.09 (s, 2H, Gln3 Hγ), 1.91 – 1.86 (m, 1H, Gln3 Hβ), 1.82 (s, 3H, Ac), 1.76 – 1.62 (m, 3H, Gln3 Hβ, X5 Hβ), 1.58 – 1.53 (m, 1H, X5 Hε), 1.39 (s, 5H, X5 Hγ Hδ Hε), 1.25 (d, J = 7.4 Hz, 6H, Ala2/Ala4 Hβ).

21B 4 mg (8% isolated based on resin loading). General analytical method A, Rt: 3.53 min. General analytical method B, Rt: 25.62 min. MS $[M+H]^+$ = 574 (found), 574 (calc.). ^{1}H NMR (500 MHz, DMSO, 298K) δ 8.45 (d, J = 7.0 Hz, 1H, NH), 8.13 (dd, J = 17.1, 6.1 Hz, 2H, NH), 8.02 (d, J = 8.7 Hz, 1H, NH), 7.24 (dd, J = 24.7, 8.4 Hz, 4H, NH), 6.75 (s, 1H, NH), 4.61 (s, 1H, Hα), 4.17 – 4.09 (m, 3H, Hα), 4.09 – 3.99 (m, 1H, Hα), 3.06 – 2.95 (m, 2H, Cys Hβ), 2.88 – 2.80 (m, 1H, X5 Hζ), 2.64 (dd, J = 13.4, 5.8 Hz, 1H, X5 Hζ), 2.16 – 2.02 (m, 2H, Gln3 Hγ), 1.91 – 1.72 (m, 7H, Ac, Gln3 Hβ, X5 Hβ), 1.63 – 1.48 (m, 3H, X5 Hγ Hε), 1.39 – 1.19 (m, 9H, Ala2/Ala4 Hβ, X5 Hδ Hε).

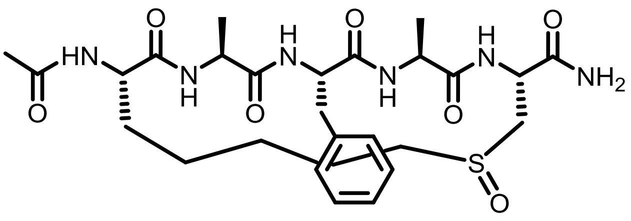

Chemical Formula: $C_{27}H_{40}N_6O_7S$
Exact Mass: 592.2679

The thiolether peptide Ac-*c*(1,5)-[X5AFAC]-Resin (0.1 mmol) was synthesized following general procedure B. After cleavage, the precipitate was dissolved in 5% H_2O_2 (5 mL) and the mixture was stirred at room temperature for 3 h. Then the solution was purified on RP HPLC.

22A 5 mg (8% isolated based on resin loading). General analytical method A, Rt: 5.26 min. General analytical method B, Rt: 31.85 min. MS $[M+H]^+$ = 593 (found), 593 (calc.). ^{1}H NMR (500 MHz, DMSO, 298K) δ 8.46 (d, *J* = 5.7 Hz, 1H, NH), 8.32 (t, *J* = 7.2 Hz, 2H, NH), 7.97 (d, *J* = 7.8 Hz, 1H, NH), 7.38 (s, 1H, NH), 7.29 – 7.14 (m, 7H, NH, Phe3 aromatic hydrogen), 4.44 (dd, *J* = 13.1, 6.8 Hz, 2H, Hα), 4.22 (d, *J* = 7.5 Hz, 1H, Hα), 4.17 – 4.09 (m, 1H, Hα), 3.96 – 3.88 (m, 1H, Hα), 3.11 (s, 2H, Cys Hβ), 2.97 – 2.89 (m, 2H, X5 Hζ), 2.87 – 2.78 (m, 2H, Phe3 Hβ), 1.80 (s, 3H, Ac), 1.59 (s, 2H, X5 Hβ), 1.47 (s, 1H, X5 Hε), 1.27 (d, *J* = 14.4 Hz, 5H, X5 Hγ Hδ Hε), 1.16 (dd, *J* = 17.9, 7.2 Hz, 6H, Ala2/Ala4 Hβ).

22B 8 mg (14% isolated based on resin loading). General analytical method A, Rt: 4.45 min. General analytical method B, Rt: 37.16 min. MS $[M+H]^+$ = 593 (found), 593 (calc.). ^{1}H NMR (500 MHz, DMSO, 298K) δ 8.46 (d, *J* = 6.7 Hz, 1H, NH), 8.24 (d, *J* = 5.8 Hz, 1H, NH), 8.16 (d, *J* = 6.4 Hz, 1H, NH), 8.03 (d, *J* = 8.8 Hz, 1H, NH), 7.28 – 7.17 (m, 7H, NH, Phe3 aromatic hydrogen), 7.03 (d, *J* = 7.8 Hz, 1H, NH), 4.66 – 4.57 (m, 1H, Hα), 4.40 (dd, *J* = 8.1, 5.0 Hz, 1H, Hα), 4.09 (dt, *J* = 14.8, 7.3 Hz, 1H, Hα), 4.01 (dd, *J* = 14.2, 7.2 Hz, 2H, Hα), 3.07 – 2.93 (m, 3H, Cys Hβ, X5 Hζ), 2.88 – 2.78 (m, 2H, X5 Hζ, Phe3 Hβ), 2.62 (dd, *J* = 13.9, 6.1 Hz, 1H, Phe3 Hβ), 1.87 (s, 3H, Ac), 1.74 – 1.63 (m, 2H, X5 Hβ), 1.47 (s, 3H, X5 Hγ Hε), 1.24 (d, *J* = 7.2 Hz, 6H, X5 Hγ Hδ, Ala Hβ), 1.09 (d, *J* = 7.3 Hz, 3H, Ala Hβ).

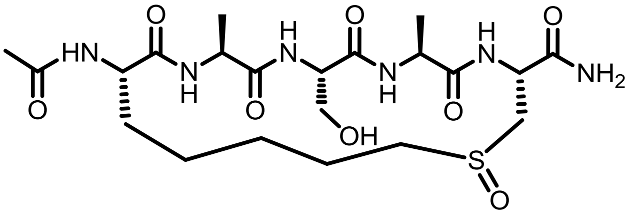

Chemical Formula: $C_{21}H_{36}N_6O_8S$
Exact Mass: 532.2315

The thiolether peptide Ac-*c*(1,5)-[X5ASAC]-Resin (0.1 mmol) was synthesized following general procedure B. After cleavage, the precipitate was dissolved in 1% H_2O_2 (5 mL) and the mixture was stirred under ice bath for 3 h. Then the solution was purified on RP HPLC.

23A 2 mg (4 % isolated based on resin loading). General analytical method B, Rt: 22.82 min. MS $[M+H]^+$ = 533 (found), 533 (calc.). 1H NMR (500 MHz, H_2O+D_2O, 298K) δ 8.42, 8.16, 8.02, 7.63, 7.25, 4.47, 4.21, 4.17, 4.16, 4.13, 4.09, 4.08, 3.95, 3.80, 3.78, 3.77, 3.66, 3.60, 3.26, 3.23, 3.14, 3.11, 3.08, 2.87, 2.86, 1.94, 1.93, 1.80, 1.78, 1.72, 1.71, 1.59, 1.57, 1.35, 1.33, 1.30, 1.29, 1.19.

23B 5 mg (9 % isolated based on resin loading). General analytical method B, Rt: 26.45 min. MS $[M+H]^+$ = 533 (found), 533 (calc.). ^{1}H NMR (500 MHz, DMSO, 298K) δ 8.47 (d, J = 6.8 Hz, 1H, NH), 8.18 (s, 1H, NH), 8.04 (dd, J = 13.4, 7.3 Hz, 2H, NH), 7.41 (d, J = 6.2 Hz, 1H, NH), 7.32 (s, 1H, NH), 7.20 (s, 1H, NH), 5.36 – 5.27 (m, 1H, Ser3 Hα), 4.53 (s, 1H, Hα), 4.18 – 4.07 (m, 3H, Hα), 4.07 – 3.99 (m, 1H, Hα), 3.64 (d, J = 6.7 Hz, 1H, Ser3 Hβ), 3.55 (s, 1H, Ser3 Hβ), 3.05 – 2.97 (m, 3H, Cys Hβ, X5 Hζ), 2.85 – 2.81 (m, 1H, X5 Hζ), 2.00 (dd, J = 14.7, 7.1 Hz, 2H, X5 Hβ), 1.99 (s, 3H, Ac), 1.81 – 1.72 (m, 2H, X5 Hε), 1.56 (s, 3H, X5 Hγ Hδ), 1.45 (s, 1H, X5 Hδ), 1.28 (d, J = 7.3 Hz, 6H, Ala2/Ala4 Hβ).

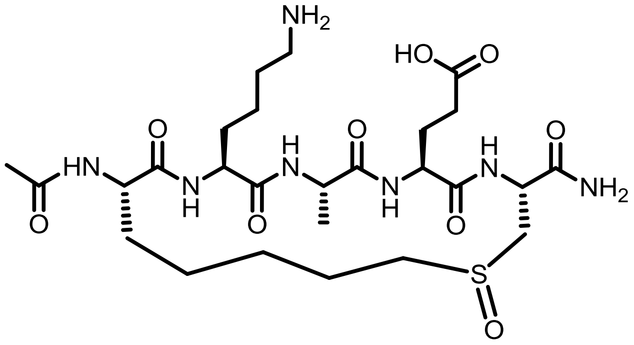

Chemical Formula: $C_{26}H_{45}N_7O_9S$
Exact Mass: 631.2999

The thiolether peptide Ac-*c*(1,5)-[X5KAEC]-Resin (0.1 mmol) was synthesized following general procedure B. After cleavage, the precipitate was dissolved in 1% H_2O_2 (5 mL) and the mixture was stirred under ice bath for 3 h. Then the solution was purified on RP HPLC.

24A 2 mg (3 % isolated based on resin loading). General analytical method B, Rt: 23.48 min. MS $[M+H]^+$ = 632 (found), 632 (calc.). 1H NMR (500 MHz, H_2O+D_2O, 298K) δ 8.57, 8.56, 8.41, 8.13, 8.12, 8.10, 8.08, 8.03, 7.54, 7.12, 4.13, 4.11, 4.10, 3.35, 3.34, 3.32, 3.31, 3.22, 3.21, 3.19, 3.18, 2.89, 2.87, 2.86, 2.84, 2.83, 2.82, 2.25, 2.23,

2.21, 2.20, 2.19, 2.17, 2.16, 2.14, 2.02, 2.00, 1.99, 1.94, 1.89, 1.86, 1.85, 1.83, 1.82, 1.73, 1.70, 1.63, 1.62, 1.58, 1.57, 1.55, 1.40, 1.39, 1.37, 1.35, 1.30, 1.29.

24B 4 mg (6 % isolated based on resin loading). General analytical method B, Rt: 23.88 min. MS $[M+H]^+$ = 632 (found), 632 (calc.).^{1}H NMR (500 MHz, DMSO, 298K) δ 12.04 (s, 1H, Glu4 COOH), 8.35 (d, *J* = 8.3 Hz, 1H, NH), 8.10 (dd, *J* = 14.9, 7.2 Hz, 3H, NH), 7.64 (s, 3H, Lys3 NH_3^+), 7.27 (t, *J* = 13.7 Hz, 2H, NH), 4.65 (d, *J* = 8.6 Hz, 1H, Hα), 4.24 – 4.18 (m, 1H, Hα), 4.17 – 4.06 (m, 2H, Hα), 3.99 (s, 1H, Hα), 3.12 – 3.03 (m, 1H, Cys Hβ), 2.98 (d, *J* = 13.3 Hz, 1H, Cys Hβ), 2.86 (s, 1H), 2.76 (d, *J* = 5.9 Hz, 2H), 2.65 (d, *J* = 13.1 Hz, 1H), 2.37 – 2.24 (m, 1H), 2.03 – 1.94 (m, 1H), 1.94 – 1.71 (m, 6H), 1.52 (s, 5H), 1.40 – 1.19 (m, 9H).

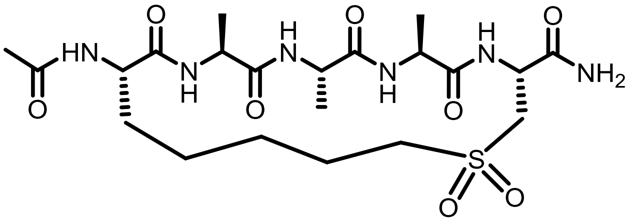

Chemical Formula: $C_{21}H_{36}N_6O_8S$
Exact Mass: 532.2315

Peptide **15** (3mg) was dissolved in acetic acid (1 mL), and then adding 50 uL 30% H_2O_2. The mixture was stirred at room temperature for 8 h. Then the solution was diluted with 3 mL water and purified on RP HPLC.

25 2 mg (65 % isolated). General analytical method A, Rt: 3.33 min. MS $[M+H]^+$ = 533 (found), 533 (calc.).^{1}H NMR (500 MHz, DMSO, 298K) δ 8.40 (dd, *J* = 12.5, 7.9 Hz, 2H, NH), 7.96 (d, *J* = 7.9 Hz, 1H, NH), 7.40 (s, 1H, NH), 7.25 (s, 2H, NH), 4.56 (d, *J* = 8.6 Hz, 1H, Hα), 4.19 (td, *J* = 15.4, 8.0 Hz, 3H, Hα), 4.00 – 3.91 (m, 1H, Hα), 3.44 (dd, *J* = 15.2, 9.5 Hz, 2H, Cys Hβ), 3.14 (dd, *J* = 17.4, 10.9 Hz, 1H, X5 Hζ), 3.09 (d, *J* = 9.1 Hz, 1H, X5 Hζ), 1.80 (s, 3H, Ac), 1.72 (s, 3H, X5 Hβ Hε), 1.40 (d, *J* = 61.6 Hz, 5H, X5 Hγ Hδ Hε), 1.29 – 1.07 (m, 9H, Ala2-4 Hβ).

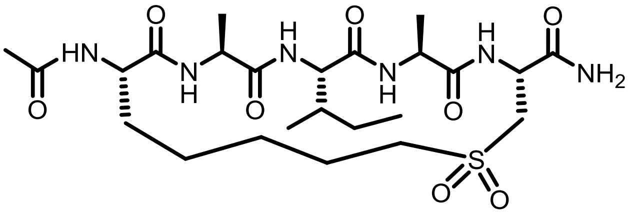

Chemical Formula: $C_{24}H_{42}N_6O_8S$
Exact Mass: 574.2785

Peptide **19** (3mg) was dissolved in acetic acid (1 mL), and then adding 50 uL 30% H_2O_2. The mixture was stirred at room temperature for 8 h. Then the solution was diluted with 3 mL water and purified on RP HPLC.

26 2 mg (65 % isolated). General analytical method A, Rt: 4.73 min. MS $[M+H]^+$ = 575 (found), 575 (calc.).^{1}H NMR (500 MHz, DMSO, 298K) δ 8.49 (d, *J* = 7.8 Hz, 1H, NH), 8.42 (d, *J* = 8.4 Hz, 2H, NH), 7.99 (d, *J* = 7.8 Hz, 1H, NH), 7.25 (d, *J* = 19.0 Hz, 2H, NH), 7.07 (s, 1H, NH), 4.59 (s, 1H, Hα), 4.26 – 4.14 (m, 3H, Hα), 3.96 (s, 1H, Hα), 3.32 (d, *J* = 14.4 Hz, 2H, Cys Hβ), 3.17 (s, 1H, X5 Hζ), 3.10 (s, 1H, X5 Hζ), 1.81 (s, 3H, Ac), 1.73 (s, 4H, X5 Hβ Hε), 1.41 (d, *J* = 52.1 Hz, 6H), 1.24 (d, *J* = 7.0 Hz, 2H), 1.07 (s, 1H,), 0.84 (dd, *J* = 16.7, 7.1 Hz, 6H).

4. SI Figures and tables

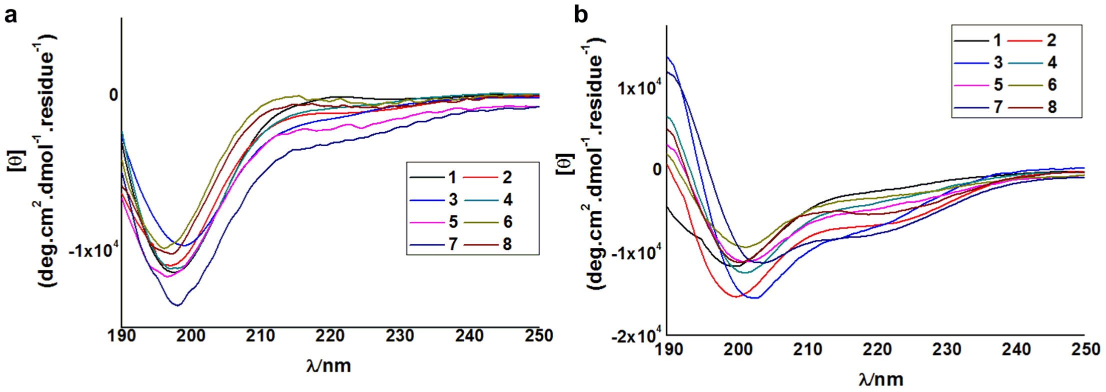

SI Figure 1. a) CD spectra of peptide **1-8** in PBS buffer (pH=7.4)[3]; b) CD spectra of peptide **1-8** in 50% TFE buffer. Peptide **1-4** = Ac-*c*(1,5)-[CAAAXn]-NH_2 n=3-6 and peptide **5-8**= Ac-*c*(1,5)-[XnAAAC]-NH_2 n=3-6.

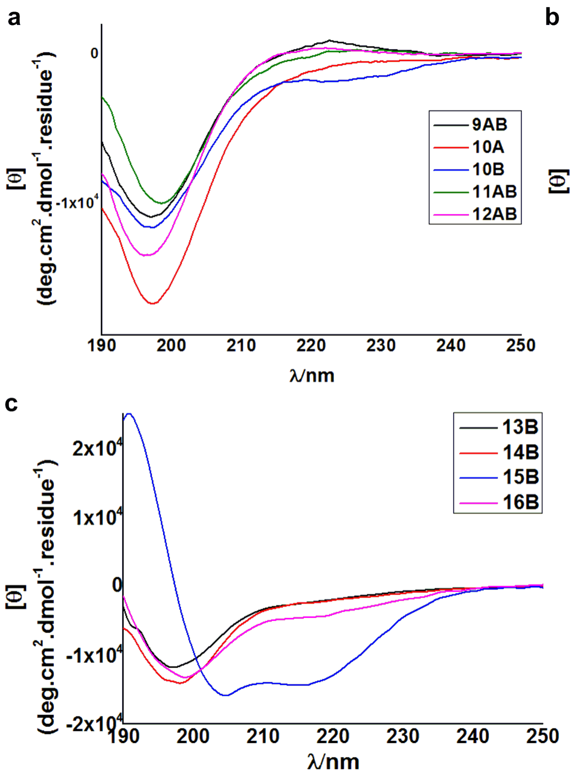

SI Figure 2. a) CD spectra of peptide **9-12AB** in PBS; b) CD spectra of peptide **13A-16A** in PBS; c) CD spectra of peptide **13B-16B** in PBS. Peptide **9AB** = Ac-c(1,5)-[C(O)AAAX3]-NH_2, **10A**, **10B** = Ac-c(1,5)-[C(O)AAAX4]-NH_2, **11AB** = Ac-c(1,5)-[C(O)AAAX5]-NH_2; **12AB** = Ac-c(1,5)-[C(O)AAAX6]-NH_2; Peptide **13A**, **13B** = Ac-c(1,5)-[X3AAAC(O)]-NH_2; **14A**, **14B** = Ac-c(1,5)-[X4AAAC(O)]-NH_2; **15A**, **15B** = Ac-c(1,5)-[X5AAAC(O)]-NH_2; **16A**, **16B** = Ac-c(1,5)-[X6AAAC(O)]-NH_2

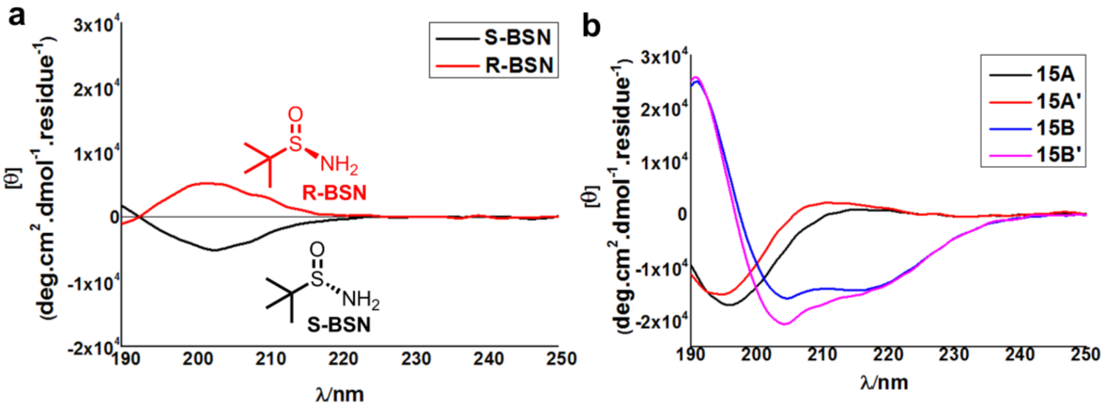

SI Figure 3. a) CD spectra of chiral sulfoxide **S-BSN** and **R-BSN**; b) **15A** and **15B** and respectively subtract **S-BSN** and **R-BSN** ones (**15A'** and **15B'**).

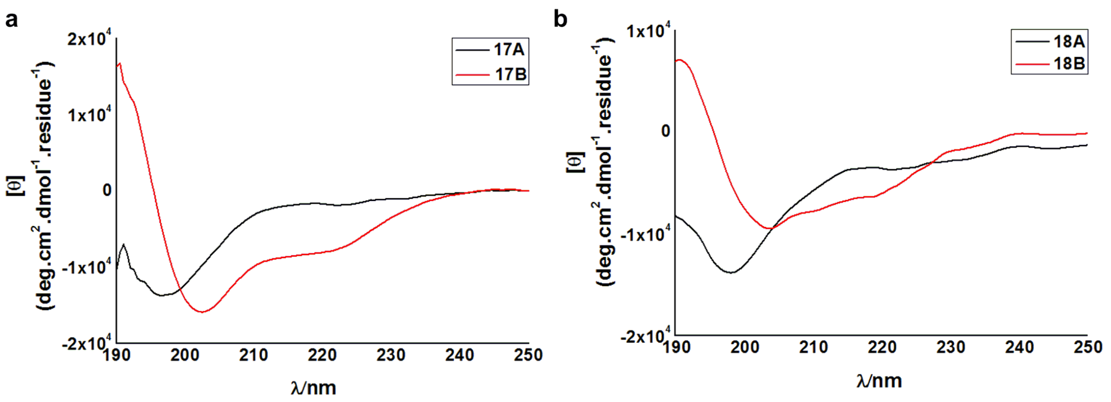

SI Figure 4. CD spectra of a) **17A** and **17B;** b) **18A** and **18B**. Peptide **17A**, **17B** =Ac-*c*(1,5)-[X4AAAC*(O)]-NH_2; **18A**, **18B** =Ac-*c*(1,5)-[X5*AAAC(O)]-NH_2.

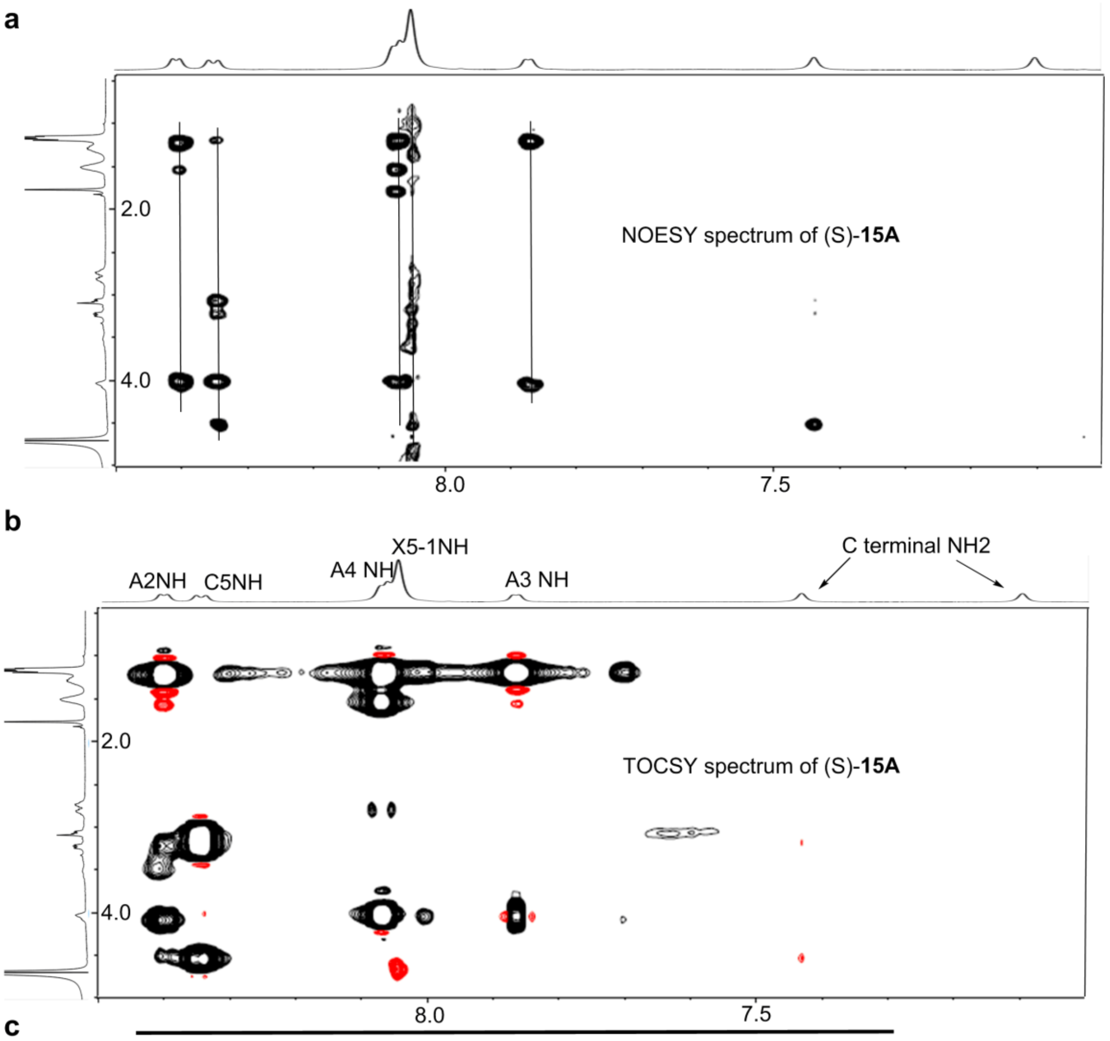

c

Residue	NH	Hα	Hβ and other sidechain hydrogens
**X5-1**	8.048	4.046	2.804 2.787 1.545 1.333 1.253
**A2**	8.401	4.089	1.221
**A3**	7.868	4.030	1.204
**A4**	8.068	4.037	1.189
**C5**	8.347	4.557	3.233 3.091

SI Figure 5. a) Section from the 500 MHz NOESY spectrum for (S)-**15A** in H_2O:D_2O (9:1). b) Fingerprint region from the 500 MHz TOCSY spectrum for (S)-**15A** in H_2O:D_2O (9:1). C) Hydrogen assigment of (S)-**15A**.

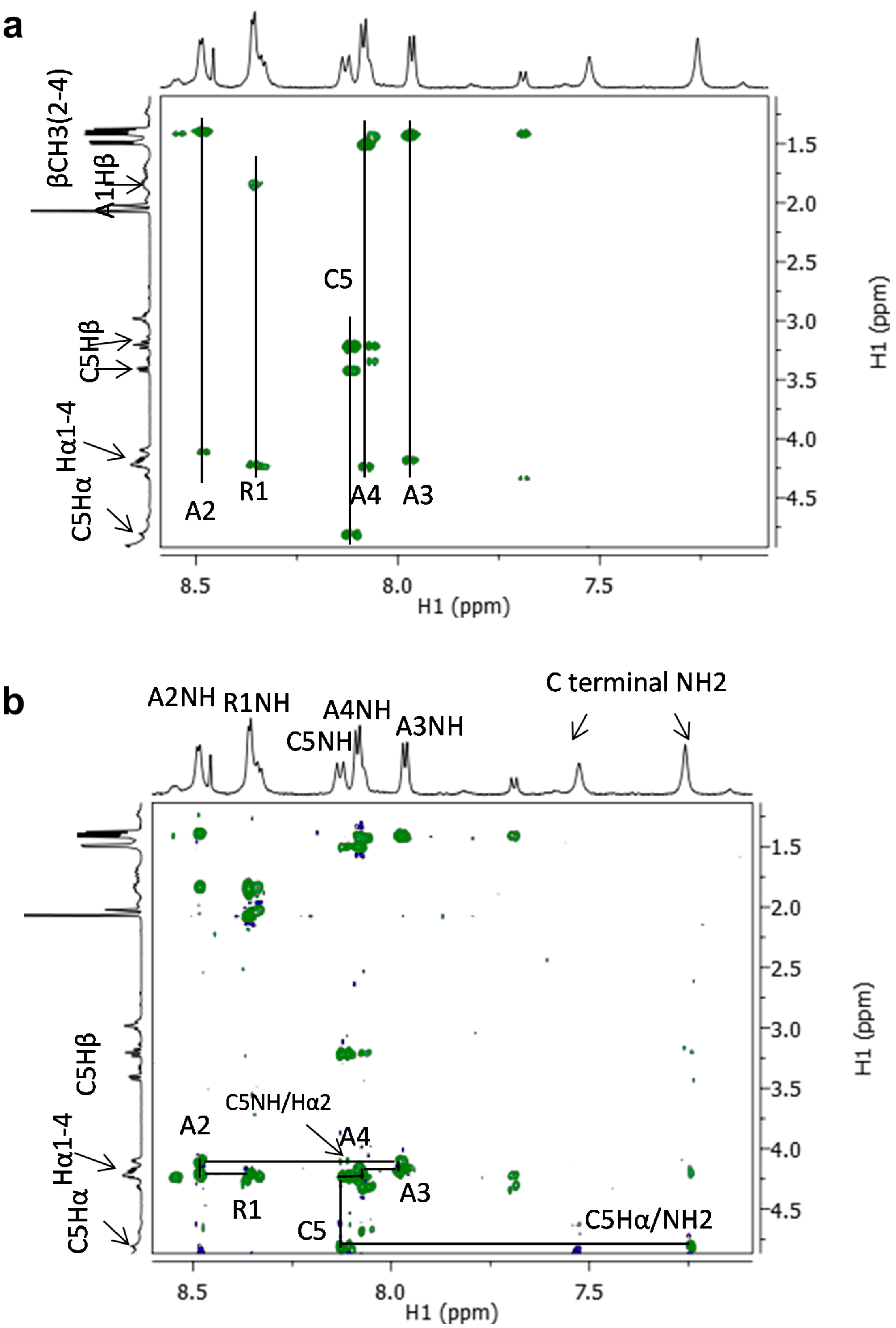

SI Figure 6. a) Section from the 500 MHz ROESY spectrum for (R)-**15B** in H_2O:D_2O (9:1) . Sequential connectivity is indicated by solid lines. Inter-residue NH-Hα cross peaks are labelled according to standard one letter amino acid codes and by their

sequence position. Long range C5NH/Hα2 cross peak was also observed. Due to the signal overlapping of Hα 1 and 4, C5NH/Hα1 and A4NH/Hα1 might be buried under C5NH/Hα4 and A4NH/Hα4. b)Fingerprint region from the 500 MHz TOCSY spectrum for (R)-**15B** in PBS:D_2O (9:1) at 283K. Spin systems are indicated by solid lines and are labelled according to standard one letter amino acid codes and by their sequence position.

SI Table 1. ^{1}H-NMR chemical shifts (δ, ppm) and $^3J_{NH\text{-}\alpha H}$ (Hz) for peptide (S)-**15A**, (R)-**15B** and (R)-**19B** in H_2O/D_2O 9:1 or PBS:D_2O 9:1. *Not determined due to peak overlap.

$^3J_{NH\text{-}\alpha H}$ (Hz)	1	2	3	4	5
(S)-**15A**	*	5.3	4.3	4.9	7.2
(R)-**15B**	3.1	3.7	5.1	5.3	8.2
(R)-**19B**	2.5	3.8	7.2	4.6	8.1

SI Table 2. Temperature dependence for amide NH and C terminus NH2 chemical shifts of(S)-**15A**, (R)-**15B** and (R)-**19B** in H_2O/D_2O 9:1 or PBS:D_2O 9:1.

Δδ/T(ppb/K)	1	2	3	4	5	NH2-1	NH2-2
(S)-**15A**	-6.3	-4.0	-3.2	-3.2	-3.2	-9.6	-9.6
(R)-**15B**	-6.3	-3.6	-8.5	-3.5	0.1	-8.0	1.4
(R)-**19B**	-6.3	-3.1	-6.6	-4.5	-0.8	-8.4	2.0

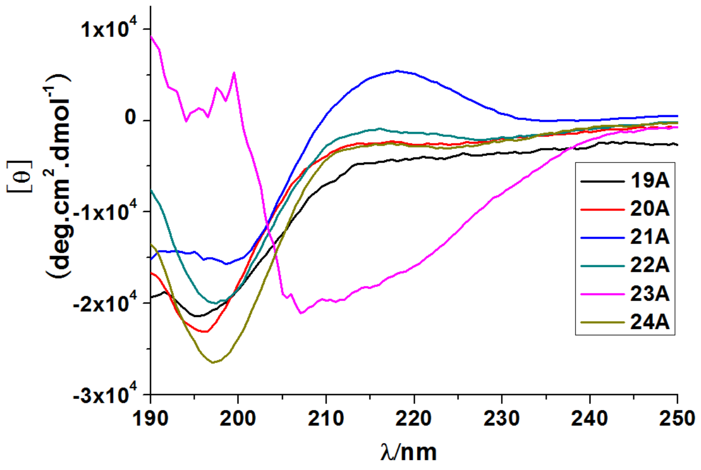

SI Figure 7. CD spectra of **19-24A**. Peptide **19A-23A**=Ac-*c*(1,5)-[X5AYAC(O)]-NH_2 Y=I, G, Q, F, S; **24A**=Ac-*c*(1,5)-[X5KAEC(O)]-NH_2

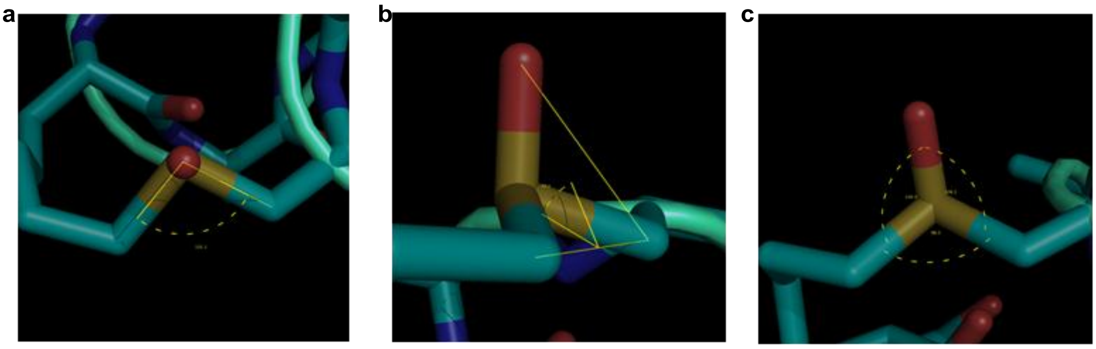

SI Figure 8. Dihedral angles of the sulfoxide center, derived from the crystal structure of peptide **19B** Ac-*c*(1,5)-[X5AIAC(O)]-NH_2. Small bond angles helps on maintaining peptide backbone constrains.

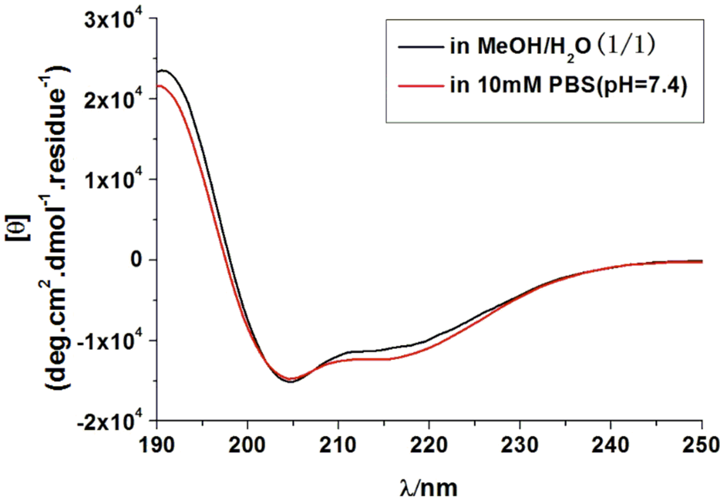

SI. Figure 9. CD spectrum of Peptide **19B** dissolved in 10mM PBS and MeOH/H_2O (1:1).

SI Table 3. Structure of peptide (R)-**19B** analyzed by TWISTER.

**Residue**	ϕ (deg)	ψ (deg)	ω (deg)	**Rise per residue, *h*(Å)**	**Residue per α-helical turn, *n***
**ideal α-helix**	-65	-40	180	1.50	3.60
**19B-helical-1**					
**X_5-1**	-61	-43	-177		
**Ala-2**	-63	-44	-177	1.40	3.72
**Ile-3**	-65	-40	-179	1.47	3.68
**Ala-4**	-65	-26	177	1.54	3.64
**Cys-5**	-109	-8	-172		
**19B-helical-2**					
**X_5-1'**	-57	-47	-178		
**Ala-2'**	-62	-42	-172	1.41	3.75
**Ile-3'**	-66	-39	180	1.44	3.72
**Ala-4'**	-72	-22	180	1.48	3.69
**Cys-5'**	-111	-10	-172		

5. Crystal data

Structure determination and Refinement. Data collection, integration, scaling and empirical absorption correction was carried out in the Rigaku CrystalClear-2.0[4] program package. The structure was solved in 1.3Å resolution by direct method using the software of *SIR2011*[5] and well refined by Full-Matrix-Least-Squares against $F^2$by*SHELXTL97*[6]. The non-hydrogen atoms were anisotropically refined and hydrogen atoms were placed at idealized positions and refined using the riding model. The absolute configuration was determined using the method of *Flack*[7]. The statistics of data collection and final refinement were shown in Table-1.

SI Table 2. The Statistics of Data collection and structure refinement

Crystal Name	Ac-*c*(1,5)-[X5AIAC(O)]-NH_2-19B-α-helix
Data collection	
Chemical formula	C24 H52 N6 O12 S
Molecular Weight	648.78
Temperature (K)	100
Space group	P1
a, b, c (Å)	9.9640 (3), 10.1650 (6), 19.2390 (11)
α β γ (°)	100.913(4), 90.944(6), 117.159(5)
V ($Å^3$)	1690.55(15)
Z Value	2
Radiation type	Cu Kα, 1.54187 Å
Crystal size (mm)	0.05 × 0.13 × 0.02
Diffractometer	Rigaku Saturn944+ (2 × 2 bin mode)
Absorption correction	multi-scan
No. ofreflections	
Measured	47745
independent	7608
observed ([I > 2σ(I)])	7544
R_{int}	0.078
(sin θ / λ)max ($Å^{-1}$)	0.562
**Refinement**	
R[F2 > 2σ(F2)], wR(F2), S	0.0833, 0.2478, 1.152
Flack parameter	0.06(4)
No. of reflections	7608
Friedel pairs	2820
No. of parameters	785
No. of restraints	3
H-atom treatment	H-atom parameters constrained

6. References:

1. Zhang, Q., Shi, X., Jiang, Y. & Li, Z. Influence of α-methylation in constructing stapled peptides with olefin metathesis. *Tetrahedron* **70**, 7621-7626 (2014).

2. Aimetti, A. A., Shoemaker, R. K., Lin, C. C. & Anseth, K. S. On-resin peptide macrocyclization using thiol-ene click chemistry. *Chem. Commun.* **46**, 4061-4063 (2010).

3. Shepherd, N. E., Hoang, H. N., Abbenante, G. & Fairlie, D. P. Single turn peptide alpha helices with exceptional stability in water. *J. Am. Chem. Soc.* **127**, 2974-2983 (2005).

4. Pflugrath, J. W. The finer things in X-ray diffraction data collection. *Acta Crystallogr D Biol Crystallogr* **55**, 1718-1725 (1999).

5. Burla, M. C. *et al*. SIR2011: a new package for crystal structure determination and refinement. *J. Appl. Crystallogr* **45**, 357-361 (2012).

6. Sheldrick, G. M. A short history of SHELX. *Acta Crystallogr* A64, 112-122 (2008).

7. Flack, H. D. On Enantiomorph-Polarity Estimation. *Acta Crystallogr.* **A39**, 876-881 (1983).

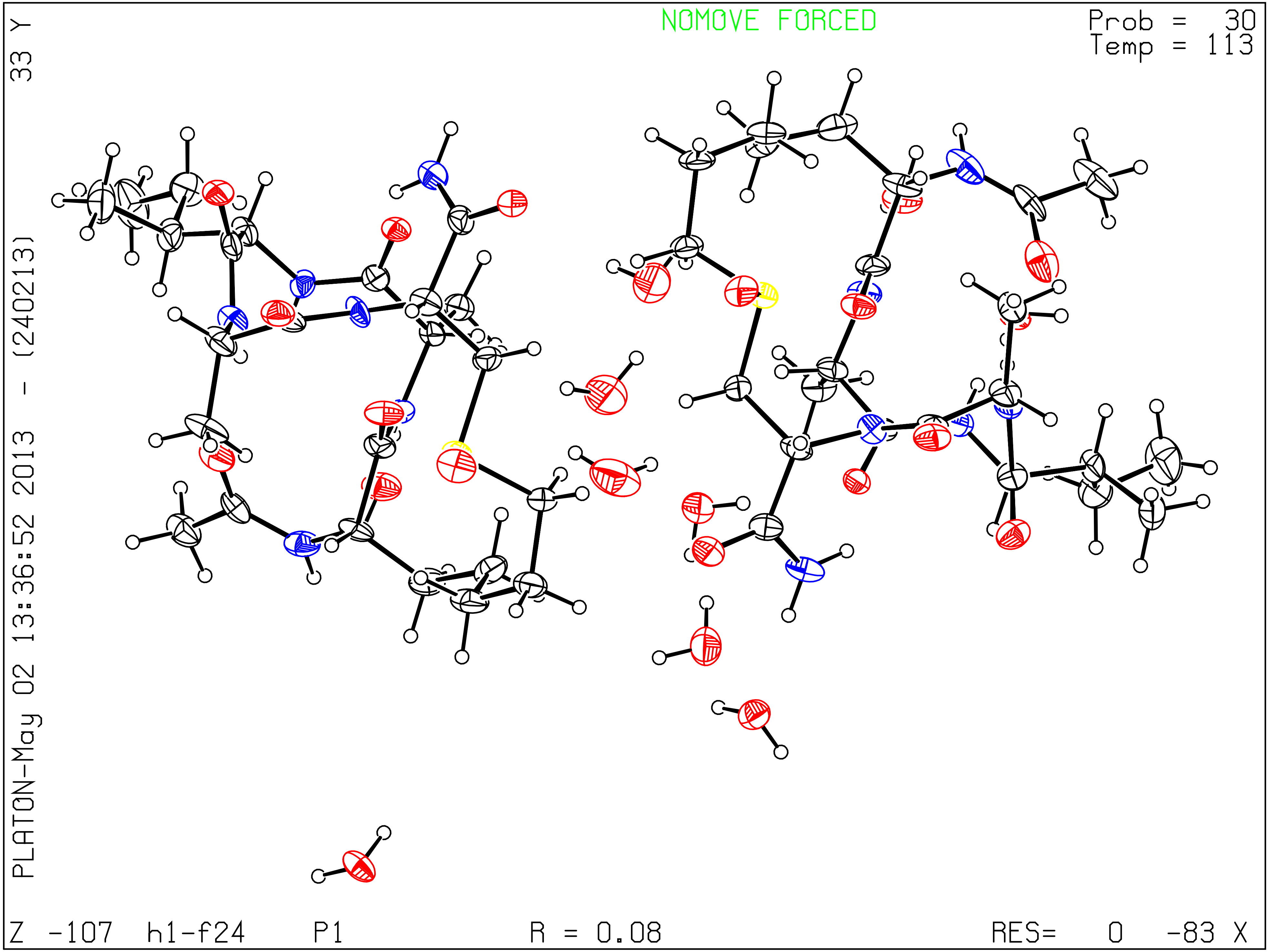
NOMOVE FORCED
Prob = 30
Temp = 113
33 Y
PLATON-May 02 13:36:52 2013 - (240213)
Z -107 h1-f24 P1 R = 0.08 RES= 0 -83 X

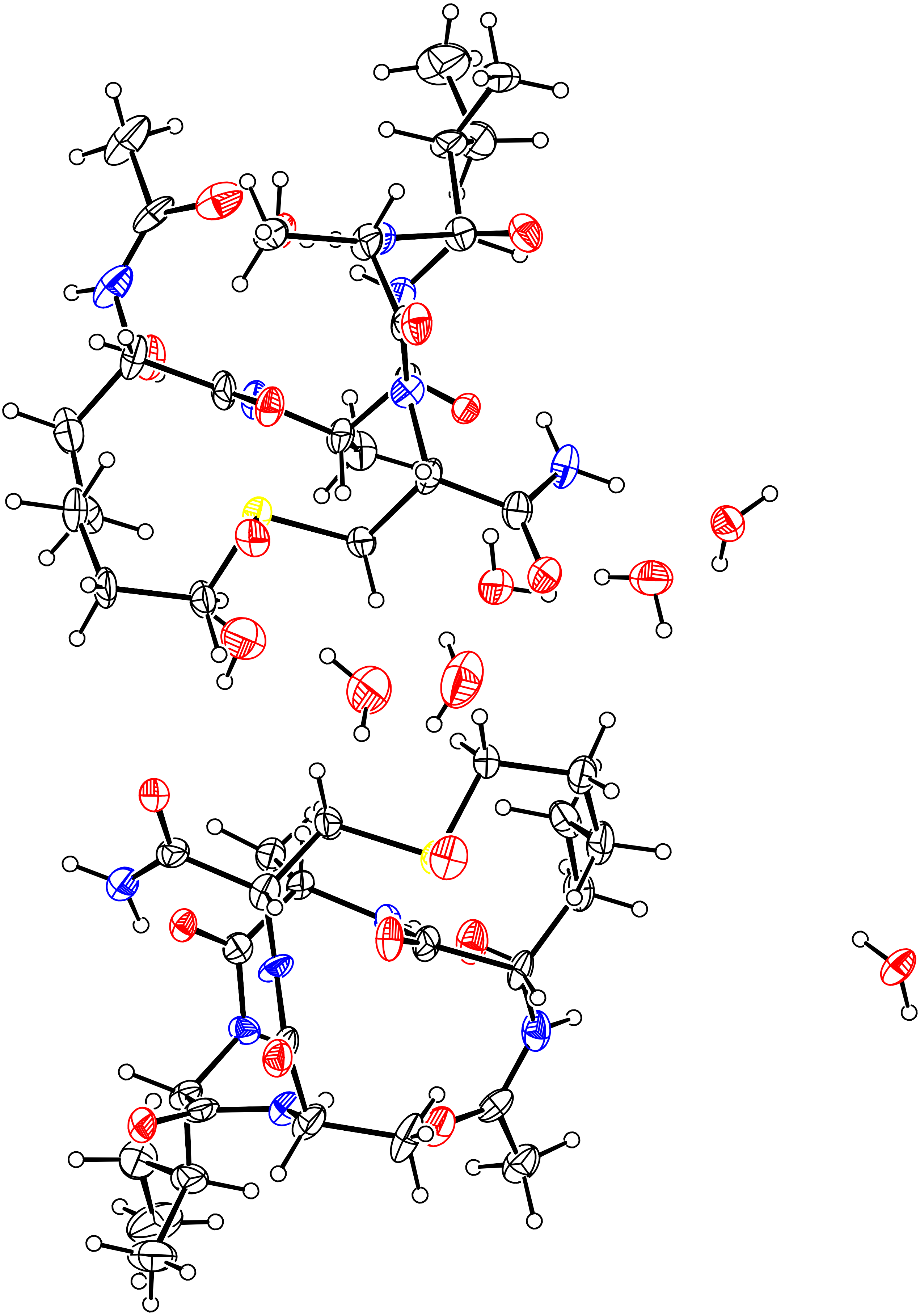

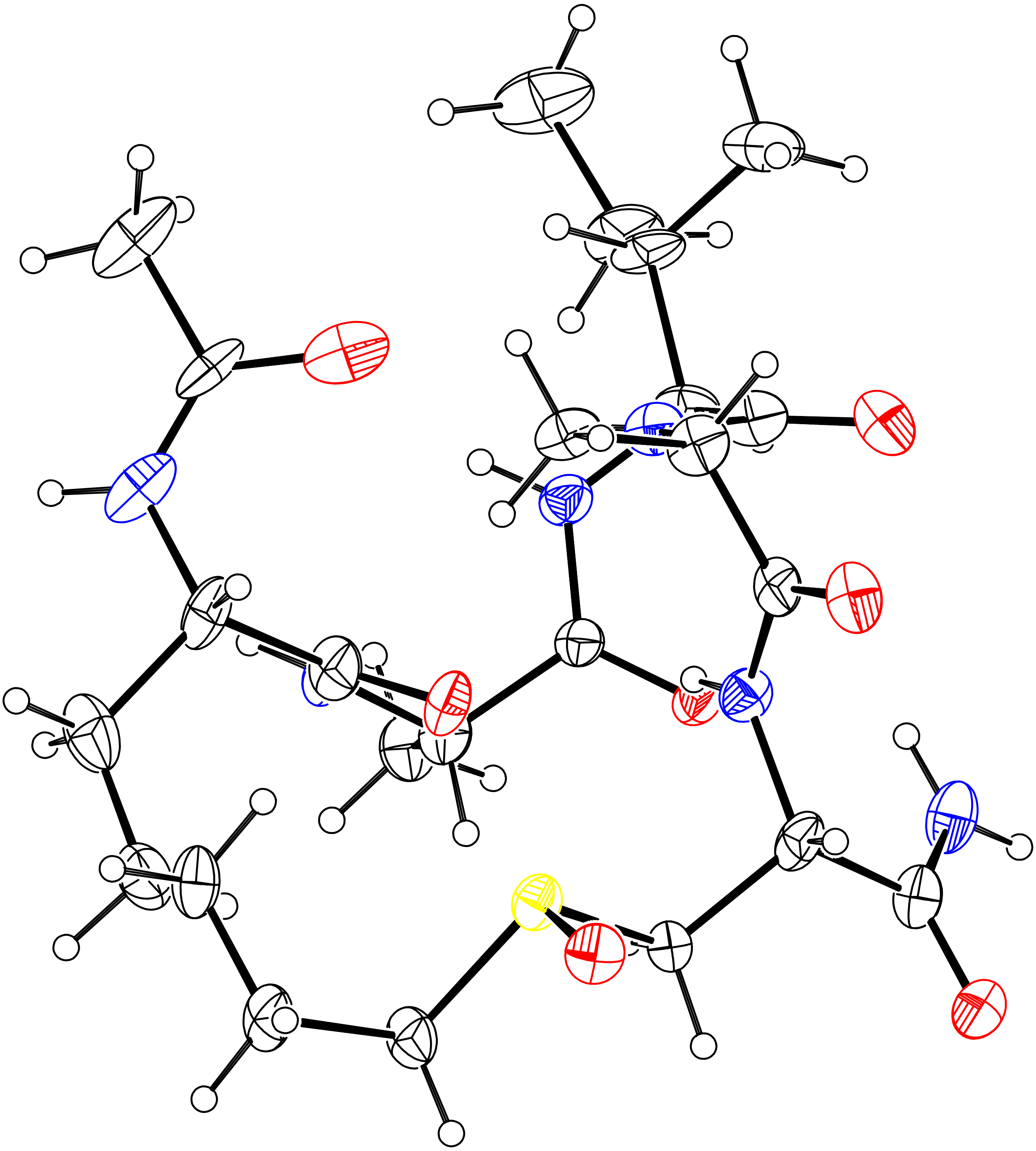

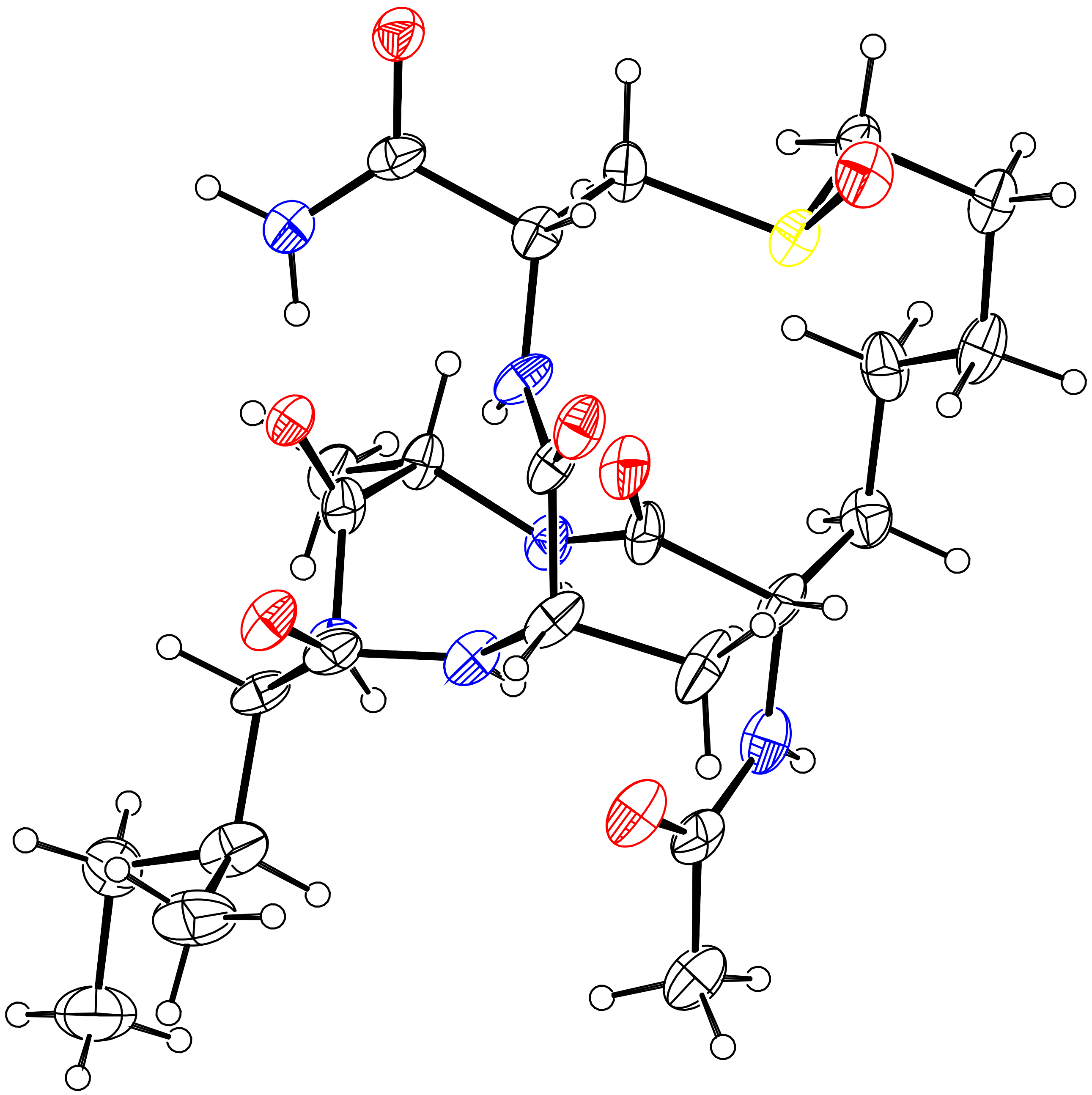

Supplement: Supplementary Information [file srep38573-s1.pdf]
